# Decoding polarity gradient enabled ultra-high lithium ion conduction

**DOI:** 10.1093/nsr/nwaf543

**Published:** 2025-11-29

**Authors:** Yuqing Chen, Aiping Wang, Yun Zhao, Wei Wang, Robert Dominko, Peitao Xiao, Peng Gao, Yan Duan, Baohua Li, Xiangming He, Jilei Liu

**Affiliations:** College of Materials Science and Engineering, Hunan Joint International Laboratory of Advanced Materials and Technology of Clean Energy, Hunan Province Key Laboratory for Advanced Carbon Materials and Applied Technology, Hunan University, Changsha 410082, China; Zhejiang Collaborative Innovation Center for Full-Process Monitoring and Green Governance of Emerging Contaminants, Interdisciplinary Research Academy, Zhejiang Shuren University, Hangzhou 310021, China; Department of materials chemistry, National Institute of Chemistry, Ljubljana SI-1000, Slovenia; Shenzhen Key Laboratory on Power Battery Safety and Shenzhen Geim Graphene Center, Tsinghua Shenzhen International Graduate School (SIGS), Shenzhen 518055, China; College of Materials Science and Engineering, Hunan Joint International Laboratory of Advanced Materials and Technology of Clean Energy, Hunan Province Key Laboratory for Advanced Carbon Materials and Applied Technology, Hunan University, Changsha 410082, China; Department of materials chemistry, National Institute of Chemistry, Ljubljana SI-1000, Slovenia; College of Aerospace Science and Engineering, National University of Defense Technology, Changsha 410073, China; College of Materials Science and Engineering, Hunan Joint International Laboratory of Advanced Materials and Technology of Clean Energy, Hunan Province Key Laboratory for Advanced Carbon Materials and Applied Technology, Hunan University, Changsha 410082, China; College of Materials Science and Engineering, Hunan Joint International Laboratory of Advanced Materials and Technology of Clean Energy, Hunan Province Key Laboratory for Advanced Carbon Materials and Applied Technology, Hunan University, Changsha 410082, China; Shenzhen Key Laboratory on Power Battery Safety and Shenzhen Geim Graphene Center, Tsinghua Shenzhen International Graduate School (SIGS), Shenzhen 518055, China; Institute of Nuclear and New Energy Technology, Tsinghua University, Beijing 100084, China; College of Materials Science and Engineering, Hunan Joint International Laboratory of Advanced Materials and Technology of Clean Energy, Hunan Province Key Laboratory for Advanced Carbon Materials and Applied Technology, Hunan University, Changsha 410082, China

**Keywords:** lithium-ion batteries, electrolyte, dielectric heterogeneity, homogenization solvation structure, low-temperature performance

## Abstract

The operational stability of lithium-ion batteries under extreme cryogenic conditions remains fundamentally constrained by solvation structure heterogeneity in conventional electrolytes, where imbalanced coordination fields between high- and low-polarity solvents exacerbate desolvation barriers and interfacial ion transport resistance. Herein, this study introduces a polarity-gradient engineering (PGE) paradigm that systematically resolves solvent polarity disparity (ΔD) through atomic-scale electronic modulation. By substituting carbon with sulfur in carbonate skeletons, an 83% reduction in dielectric heterogeneity is reached (Δε = 17.1 vs. 86.6 in carbonates), enabling balanced Li⁺ coordination among cyclic/linear sulfites and anions. This homogenized solvation feature significantly accelerates desolvation kinetics (34.97 kJ·mol⁻^1^ activation energy vs. 79.1 kJ·mol⁻¹ in carbonates) and promotes the formation of LiF-rich interphase. Benefiting from these, the optimized electrolyte demonstrates liquid operation down to −110°C with 1 mS·cm⁻^1^ at −80°C, thus enabling 450 Wh·kg^−1^ LiCoO_2_/Li pouch cells to perform stable cycling at −20°C with 81% capacity retention over 400 cycles, with 73% of room-temperature capacity at −60°C. The homogeneous solvation structure intrinsically couples thermodynamic stability with accelerated interfacial kinetics, revealing a paradigm for extreme-condition energy storage. This study pioneers a universal design framework that decouples the trade-off between desolvation barriers and ion mobility, delivering an atomic-scale blueprint for cryogenic batteries.

## Teaser

This study pioneers an atomic swap of sulfur for carbon in electrolyte solvents to creates a homogenized coordination environment that unlocks high-performance lithium-ion batteries at ultra-low temperatures.

## INTRODUCTION

The operational frontiers of lithium-ion batteries are being redefined by emerging energy storage demands in extreme environments, from polar research stations to interplanetary probes. Yet their low-temperature failure under −30°C—manifested as 60%–80% capacity loss compared to room temperature—exposes fundamental limitations in electrolyte engineering [1–4]. Three interlinked failure mechanisms have been identified: solvent crystallization-induced ionic transport paralysis [5–7]; desolvation-dominated kinetic bottlenecks [8–10]; and thermally vulnerable interfacial ion migration [11–14]. Among these, the desolvation energy barrier (∼50–70 kJ·mol⁻^1^) dwarfs those of interfacial (∼20 kJ·mol⁻^1^) and bulk-phase transport (∼5 kJ·mol⁻^1^) [15–17], establishing solvent-sheath restructuring as the master key to unlock cryogenic battery performance.

Recent advances in weakly solvating electrolytes have established two dominant paradigms for cryogenic lithium batteries: (i) employing low donor-number (DN) solvents to establish loosely coordinated structures [16,18–21]; and (ii) leveraging localized high-concentration electrolytes (LHCEs) to amplify anion-involved solvation [8,22–24]. Xu *et al.* [2] pioneered ‘soft solvents’ with moderate dielectric constants (ε > 5) and low DN (<10), exemplified by methyl difluoroacetate (MDFA) and 2,2-difluoro-2-(fluorosulfonyl) acetate (MDFSA). This design facilitates Li-salt dissociation with desolvation kinetics, enabling 360 cycles with 80% capacity retention for 4.5 V NCM811/Gr pouch cells at −30°C. Similarly, Qin *et al.* [18] mitigated Li-propylene carbonate (PC) coordination through fluorobenzene (FB) dilution, simultaneously enhancing desolvation dynamics and graphite compatibility. For the LHCE strategy, Fan *et al.*’s [8] breakthrough using a non-polar fluoroether co-solvent strategy reshaped ion–solvent interactions, achieving ultrawide-temperature operation (−125°C to 70°C), with 55% capacity retention at −85°C for NCA/Li cells, which is a milestone in electrolyte engineering.

Despite the promise of weakly solvating electrolytes in lowering desolvation barriers and constructing inorganic-rich interphases for cryogenic operation, their practical implementation is fundamentally constrained by overlooked solvation disparity stemming from solvent polarity heterogeneity. Thermodynamically, dielectric heterogeneity (∆ε) in mixed solvents promotes non-ideal solvation clustering and localized polarity domains, distorting Li⁺ transport pathways [25,26]—a phenomenon analogous to microphase segregation in polymer electrolytes [27–30]. While the spatial scale of heterogeneity differs between liquid and polymer electrolytes, the underlying thermodynamic drive for polarity-driven segregation is universal. Kinetically, the polarity gradient dictates that high-dielectric-constant solvents preferentially coordinate with Li⁺ and are thereby transported to the electrode interface. This leads to a compositionally distorted electric double-layer (EDL) architecture [25,26,28,31–33], thereby perturbing desolvation–deposition coupling (for more details, see Supplementary data). These effects impose intrinsic limitations on existing strategies: for low-DN solvents (e.g. FB), the intrinsic dielectric–donor correlation creates a physicochemical paradox—weakened coordination strength inevitably sacrifices Li-salt dissociation efficiency [16], as evidenced by aggravated ion pairing. This duality ultimately caps the ‘dissociation–coordination’ optimization ceiling. In LHCE systems, while non-polar diluents reduce viscosity, their polarity mismatch with polar solvents induces cryogenic phase separation [34] and localized concentration fluctuations. Moreover, diluent-surface adsorption competition destabilizes EDL configurations [35], accelerating interfacial side reactions.

**Figure 1.**
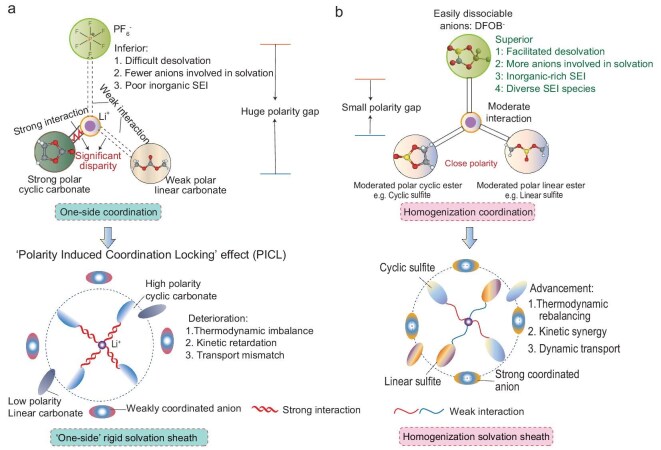
Molecular PGE-induced solvation structure. (a) ‘One-side’ solvation sheath of traditional carbonate electrolytes. (b) Homogenization solvation sheath proposed in this work.

These challenges stem from an overlooked fundamental principle in conventional electrolyte design: the solvation synergy of mixed-solvent polarity. Taking the extensively studied ethylene carbonate/dimethyl carbonate (EC–DMC) carbonate system as an example, the extreme dielectric contrast [Δε = 86.5 between EC (ε = 89.6) and DMC (ε = 3.1)] creates a polarized coordination environment where Li⁺–EC binding energy (−1.82 eV) overwhelmingly dominates over Li⁺–DMC (−0.67 eV). This imbalance forces anisotropic coordination fields, forming a rigid ‘one-side’ solvation sheath with strong-polar solvents in inner coordination spheres and weak-polar components on the periphery (Fig. 1a, Fig. S1a). Crucially, this structural dichotomy becomes catastrophic at low temperatures: inner-sphere strong coordinators demand excessive desolvation energy, while outer-sphere weak coordinators lose kinetic fluidity to compensate transport dynamics. The resulting ‘polarity-induced coordination locking’ (PICL) effect manifests 3-fold deterioration: (i) thermodynamic imbalance excluding anions from solvation shells, hindering inorganic-rich solid–electrolyte interphase (SEI) formation; (ii) increased kinetic barriers due to higher rigid-sheath molecular rearrangement energy; and (iii) transport mismatch from dielectric heterogeneity-induced tortuous Li⁺ migration pathways.

To address these issues, this study pioneers a polarity-gradient engineering (PGE) paradigm to resolve the solvation dichotomy. By strategically compressing dielectric heterogeneity (Δε) through atomic-scale electronic modulation, we establish balanced Li⁺ coordination among high/low-polarity solvents and anions, ultimately forging a homogenized solvation feature (Fig. 1b, Fig. S1). Specifically, substituting carbon with sulfur in carbonate skeletons leverages sulfur’s larger covalent radius (1.04 vs. 0.76 Å) and delocalized electron cloud (Figs S2 and S3), reducing Δε from 86.6 (EC–DMC) to 17.1 [ethylene sulfite (ES)/dimethyl sulfite (DMS)]. This attenuated polarity gradient equilibrates Li⁺–solvent binding energies (ES: −1.21 eV; DMS: −0.89 eV), triggering tripartite coordination competition among strong- and weak-polar solvents and anions. The PGE strategy delivers three decoupled mechanisms: (i) thermodynamic rebalancing: weakened preferential coordination with strong-polar solvents to accelerate desolvation; (ii) kinetic synergy: enhanced anion/weak-solvent participation to drive inorganic-rich interphase formation; and (iii) dynamic transport: self-optimized solvation microdomains for superior ionic conductivity. Experimental validation demonstrates that PGE-driven sulfite-based electrolytes enable 85% capacity retention over 300 cycles for LiCoO_2_ (LCO)/Li pouch cells at −20°C. Beyond empirical design conventions, this work establishes a ‘polarity gradient–solvation homogeneity–interfacial kinetics’ framework. By systematically integrating these factors, the framework provides transformative insights between desolvation barriers and ionic mobility at low temperatures.

## RESULTS AND DISCUSSION

### Rational design of polarity gradient-driven homogenization solvation

**Figure 2.**
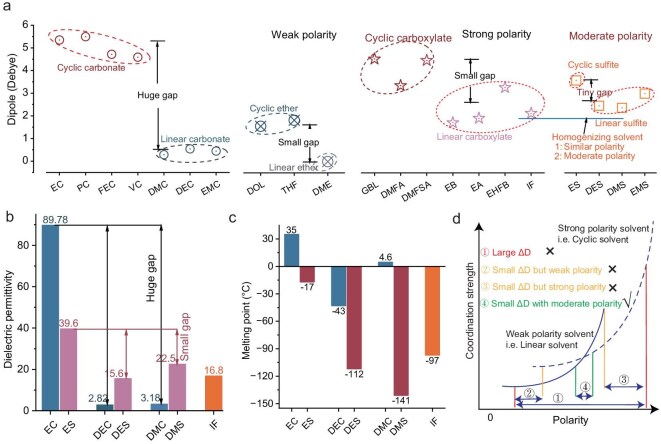
Principles for solvent screening of homogeneous solvation structure electrolyte. (a) Dipole comparison of different types of organic solvents whose full name corresponding to the abbreviation were presented in the supplementary data. (b) Comparison of dielectric constants between sulfite and carbonate solvents. (c) Melting points of sulfites and carbonates. (d) The variation of polarity as a function of coordination; solid and dashed lines represent weak- and strong-polarity solvents, respectively.

Mitigating polarity disparity (ΔD) optimally suppresses dielectric non-uniformity (Δε), driven by their causal polarity–dielectric correlation. Systematic density functional theory (DFT) screening of dipole moments across carbonate, ether, carboxylate and sulfite solvents (Fig. 2a) reveals critical design principles for homogenized solvation features: (i) minimized ΔD between cyclic/linear counterparts as possible for balanced coordination; and (ii) moderate polarity to simultaneously ensure sufficient Li-salt dissociation and easy desolvation. Sulfite-based systems emerge as optimal candidates, exhibiting intrinsically small cyclic/linear ΔD and moderate polarity, leading to minimal dielectric contrast (Δε = 17.1 vs. 86.6 in carbonates counterparts, Fig. 2b) while maintaining favorable dielectric strength. Despite structural parallels between sulfite and carbonate esters, the sulfur–carbon substitution in carbonate frameworks provokes a pivotal question: what is the underlying mechanism driving such drastic polarity modulation between linear/cyclic esters? The answer comes from atomic-scale structural investigation. Sulfur substitution in the carbonate backbone expands covalent radius (1.04 vs. 0.76 Å for carbon) and delocalizes electron density at S=O bonds (Figs S2 and S3), mitigating steric and electronic disparities between cyclic/linear conformers. The higher atomic polarizability of sulfur (3.0023 × 10⁻^24^ cm^3^) versus C (1.2432 × 10⁻^24^ cm^3^) fundamentally attenuates the electrostatic potential contrast in ES (63.70 kcal·mol⁻^1^) compared to EC (75.22 kcal·mol⁻^1^), rationalizing its weaker solvating power and the resulting homogenized coordination environment. Furthermore, the larger covalent radius of sulfur introduces greater molecular asymmetry and steric hindrance in sulfites, which effectively suppresses their crystallization tendency and results in addressing liquid phase limitations—ES–DMS solvents demonstrate dramatically depressed melting points (−17°C/−141°C vs. 35°C/4.6°C for EC–DMC, Fig. 2c). This expanded liquid phase range is a critical prerequisite for maintaining high ionic conductivity and facilitating fast ion transport under cryogenic conditions. This design principle, which utilizes dielectric heterogeneity (Δε) as a screening tool, focuses on achieving macroscopic solvent compatibility and minimized polarity contrast. It is important to note that for predicting precise local coordination, molecular-level parameters such as DN are more appropriate [36]. To operationalize the molecular polarity-gradient framework, we established critical design criteria through non-linear correlation analysis between solvent polarity and coordination strength (Fig. 2d). The simulated response profiles (solid line: high-polarity solvents; dashed line: low-polarity counterparts) define an optimal ΔD window (green zone) where mixed solvents simultaneously achieve minimized polarity contrast and moderate dielectric strength. This ΔD-constrained selection protocol provides quantitative guidance for electrolyte formulation, ensuring balanced solvation while avoiding extreme polarization effects.

The elevated redox activity of sulfites [highest occupied molecular orbit (HOMO)–lowest unoccupied molecular orbit (LUMO) analysis, Fig. S4] necessitates strategic stabilization against interfacial decomposition. We introduced isobutyl formate (IF)—a short-chain carboxylate with low viscosity, depressed melting point and anode film-forming capability—into the sulfite system. IF exhibits exceptional compatibility, demonstrating negligible polarity contrast with sulfites (Fig. 2a, Fig. S3) while asymmetrically modulating DMS–ES polarity to further homogenize solvation environments (ΔD from 1.20 to 0.86, Fig. S5). Lithium bis(oxalato)borate (LiDFOB) was prioritized over LiPF_6_ due to its enhanced dissociation efficiency and superior ion transference number, effectively mitigating concentration polarization under cryogenic conditions. The optimized formulation (1 M LiDFOB in ES–DMS–IF [2:4.5:3.5 v/v)] synergistically integrates these design principles.

### Solvation features and ion transport mechanisms

**Figure 3.**
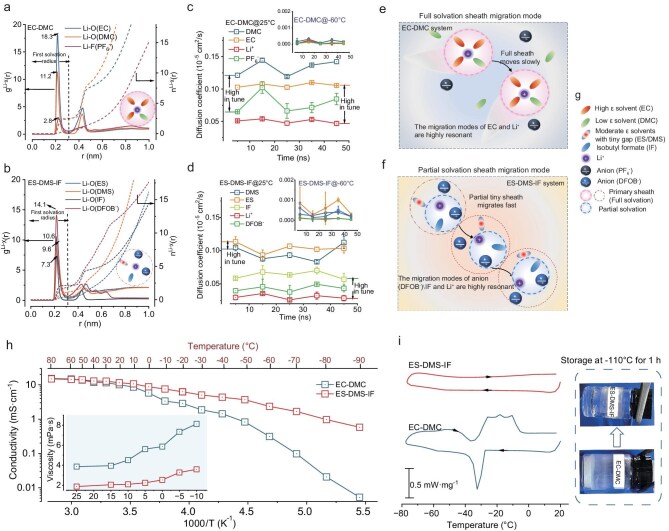
Unlocked solvated structure and proposed ion transport mechanism. (a and b) The calculated radial distribution functions [g(r)] and coordination numbers [n(r)] for EC–DMC electrolyte (a) and ES–DMS–IF electrolyte (b) based on MD simulations. (c and d) Variation of diffusion coefficients with time for various solvents and ions in EC–DMC (c) and ES–DMS–IF electrolyte (d) calculated from the RMSD. (e and f) Solvation migration mode for EC–DMC (e) and ES–DMS–IF electrolytes (f). (g) The legend of the solvation structure components. (h) Temperature-dependent ionic conductivity. (i) DSC curve of the above electrolytes and the optical photos of electrolyte after storage at −110°C for 1 h.

Multi-dimensional validation of the proposed polarity-gradient strategy combines molecular dynamics (MD) simulations and spectroscopic characterization. The solvation structures were decoupled by performing MD simulations (Fig. S6) and calculating the radial distribution functions (RDFs) [g(r)] and coordination numbers [n(r)] of Li^+^ in each case (Fig. 3a and b, Fig. S6). In general, the dominant g(r) peak for each solvent appeared at ∼2.25 Å, corresponding to the radius of the first solvation shell. Conventional EC–DMC electrolyte shows EC-dominated Li⁺ coordination [g(r)_Li-O (EC)_ = 18.3 vs. g(r)_Li-O (DMC)_ = 6.7], leading to an asymmetrical solvation sheath (Fig. 3a), with a solvation structure consisting of 67.66% solvent-separated ion pairs (SSIPs), 14.01% contact ion pairs (CIPs) and 5.80% aggregates (AGGs) (Fig. S43). In contrast, the sulfite-based system demonstrates balanced coordination with dominant anion participation [g(r)_Li-O (DFOB)_ = 14.1] and equivalent solvent contributions [g(r)_Li-O (ES) _= 10.6, g(r)_Li-O (DMS) _= 9.6, g(r)_Li-O (IF)_ = 7.3], confirming a homogeneous solvation feature (Fig. 3b). The designed electrolyte presented a typical solvation structure with a statistical ratio of ∼1.81:1.11:2.15:0.41 for DFOB:DMS:IF:ES. Comparative analysis with the single-solvent (1 M LiDFOB–ES, which has been reported in the literature [37]) and binary (1 M LiDFOB–ES–DMS) systems reveals a progressive structural evolution: from ES-dominated coordination (ES:DFOB⁻ = 3.70:1.70) to DMS-dominated (DMS:ES:DFOB⁻ = 3.55:1.58:0.90), and finally to a balanced, anion-enhanced solvation structure in the ternary electrolyte (IF:DMS:ES:DFOB⁻ = 2.15:1.32:0.43:1.94) (Fig. S44). This sequence confirms that the multi-solvent design under the PGE strategy is essential for achieving a homogenized and functionally superior solvation environment, rather than the single-solvent system which, despite eliminating dielectric heterogeneity, fails to optimize coordination thermodynamics and interfacial chemistry. Complementary spectroscopy analysis (Figs S7–S10, Tables S1 and S2) confirms the theoretical predictions. Distinct from carbonate electrolytes dominated by EC–Li⁺ coordination, sulfite-based systems exhibit spectral equivalency between ES and DMS. The Fourier transform infrared spectroscopy (FTIR) peak at 665/673 cm^−1^ shows the overlapping O–S–O stretching vibrations of ES and DMS (Figs S7 and S8, Table S1). The Raman peak located at 750 cm^−1^ is the superimposed C–S stretching modes of ES and DMS, and peaks within 915–1020 cm^−1^ represent the coupled CH_2_–CH_3_ rocking vibrations of both ES and DMS (Figs S9 and S10, Table S2). This vibrational signature parity directly evidences comparable Li⁺ solvation contributions from ES and DMS, experimentally validating the homogeneous solvation structure. Also, the spectral superposition phenomenon systematically demonstrates equivalent coordination competitiveness between cyclic/linear sulfites, conclusively supporting the proposed solvation feature (schematically illustrated in Fig. 3a and b). Furthermore, DFOB⁻ anions predominantly coordinate directly with Li⁺ in the inner solvation shell, which significantly lowers their LUMO energy level and thereby facilitates preferential reduction, presumably benefiting the formation of a LiF-rich interphase.

Furthermore, MD simulations reveal distinct ion migration patterns through mean squared displacement (MSD) analysis (Fig. 3c and d, Figs S11–S14). In carbonate systems, EC exhibits synchronized diffusion with Li⁺, while DMC exhibits synchronous motion with PF_6_⁻ (Fig. 3c), indicating rigid solvation-sheath migration that impedes ion mobility (Fig. 3e). Conversely, the sulfite-based electrolyte demonstrates decoupled transport dynamics: Li⁺ migration correlates strongly with DFOB⁻ anions and IF solvent, while ES–DMS shows transport separation diffusion profiles with Li^+^. This anion–IF–Li^+^ cooperative transport mechanism enhances ion mobility by reducing the effective solvation radius during Li⁺ migration (Fig. 3f), enabling exceptional low-temperature conductivity and viscosity (0.97 mS·cm⁻^1^ at −80°C and 3.59 mPa·s at −10°C, Fig. 3h, Fig. S15, Tables S7 and S8) without phase transition below −80°C (DSC-validated, Fig. 3i, Fig. S16). Consequently, the sulfite-based electrolyte with IF shows more excellent ion transport kinetics. Eventually, the liquid-state integrity maintained at −110°C (optical photos, Fig. 3i, Fig. S17) underscores the formulation’s cryogenic viability. Notably, the single sulfite solvent system (1 M LiDFOB–ES) was supplementarily conducted ion conductivity and viscosity tests to verify the influence of complete eliminating dielectric heterogeneity; however, it shows that the single sulfite solvent system performed inferiorly to the binary and optimized ternary electrolytes with highest viscosity (10.16 mPa·s) and smallest conductivity (2.78 mS·cm^−1^) under −20°C (Fig. S36), it demonstrates that the PGE strategy aims not only to minimize ∆ε, but also to optimize the overall solvation thermodynamics, transport dynamics and interfacial stability through a balanced multi-solvent formulation. Also, this explains the ionic conductivities of these electrolytes being predominantly determined by the viscosity over the dielectric constant.

### Interfacial optimization via homogeneous solvation

**Figure 4.**
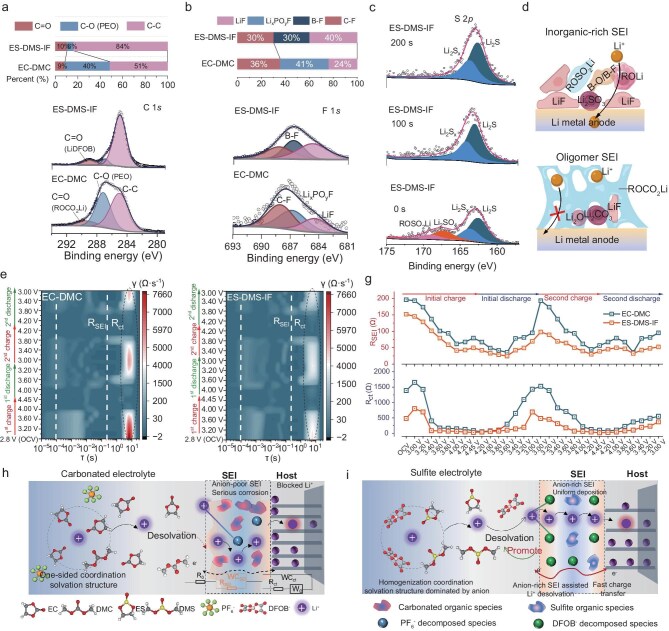
Properties of SEIs. (a–c) XPS profiles of the Li anode after three formation cycles with different electrolytes: O 1*s* (a), F 1*s* (b), and the depth profile of S *2p* (c). (d) Schematic diagram of interface component distribution. (e and f) *In situ* DRT data representing two cycles of LCO/Li with EC–DMC and ES–DMS electrolytes. (g) The corresponding fitting impedance versus voltage profiles of SEI impedance R_SEI_ and charge transfer impedance R_ct_. (h and i) Schematic diagram of ion transport in electrochemical processes for EC–DMC (h) and ES–DMS–IF electrolyte (i).

To elucidate the low-temperature interfacial modification effects of the proposed electrolyte design, X-ray photoelectron spectroscopy (XPS) analysis was performed on cycled Li metal anodes (3 cycles) (Fig. 4a–c, Figs S18 and S19, Table S3). In the EC–DMC electrolyte, the organic interfacial components predominantly originated from decomposition of the highly polar EC solvent, yielding PEO (–CH_2_–CH_2_–O–) and alkyl lithium carbonates (ROCO_2_Li) (total 49%), corresponding to C–O (286.82 eV) and C=O (288.98 eV) signals [38–40], respectively (Fig. 4a). In contrast, the ES–DMS sulfite-based electrolyte exhibited markedly reduced C–O (12%) and C=O (6%) contents (Fig. S18a), where the C=O component arose not from solvent decomposition but from LiDFOB salt degradation. Upon introducing the IF co-solvent, the C–O content in ES–DMS–IF further decreased (6%) while C=O increased (10%) (Fig. 4a, Fig. S18a), indicating suppressed reductive decomposition of the primary solvent and enhanced anion participation in solvation and SEI formation. This aligns with the F 1*s* spectra [35,41], whereas LiF content on the Li anode cycled in ES–DMS–IF (40%) exceeded that in ES–DMS (24%) (Fig. 4b, Fig. S18b). This is consistent with the LiF proportion in the Li 1*s* spectrum [42,43], which shows ES–DMS–IF (50%) > ES–DMS (30%) > EC–DMC (24%) (Fig. S19a). Notably, both sulfite-based systems exhibited higher LiF content than the carbonate-based system, with LiF solely derived from salt decomposition [44], confirming that homogeneous solvation promotes anion-derived inorganic SEI formation. LiF, a well-documented ion-conductive inorganic component, facilitates interfacial ion transport [45,46]. O 1*s* analysis [47,48] further revealed that ES–DMS–IF promoted DMS-derived ROLi formation compared to ES–DMS (Fig. S18c). ROCO_2_Li was exclusive to carbonate systems, while Li_2_CO_3_ originated from EC (carbonate electrolyte) or LiDFOB (sulfite system), with higher Li_2_CO_3_ content in ES–DMS–IF (78% vs 73% in ES–DMS), corroborating IF-enhanced LiDFOB decomposition. This agrees with the elevated B–O content (67%) in B 1*s* spectra [49,50] for ES–DMS–IF (Fig. S19b). S 2*p* analysis [51,52] further demonstrated that IF incorporation facilitated Li_2_S/LiS_x_ inorganic sulfur species formation while suppressing ES-derived ROSO_2_Li/Li_2_SO_4_ organics (Fig. S19c), suggesting preferential sulfur–inorganic decomposition pathways under homogeneous solvation. In addition, the S 2*p* depth profiling indicates a transition from mixed organic sulfites and inorganic sulfides at the surface to a purely inorganic Li_2_S-dominated environment in the inner SEI (Fig. 4c, Fig. S39d). The XPS depth profiling also reveals a definitive shift from solvent-dominated to anion-derived interfacial chemistry. The C 1*s* spectrum shows suppression of organic components (C–O/C=O) and the concomitant emergence of C–F species (Fig. S39a). Crucially, the F 1*s* spectrum demonstrates a marked increase in LiF intensity in the inner SEI region, alongside emerging B–F and C–F species (Fig. S39b). The B 1*s* spectra confirm the pivotal role of LiDFOB decomposition, showing a pronounced strengthening of inorganic B–O and B–F species with depth (Fig. S39c). Collectively, the ES–DMS–IF electrolyte forms an inorganic-rich SEI (LiF/Li_2_CO_3_/B–O/B–F/LiS_x_) on Li metal, enhancing interfacial stability, ion transport and side-reaction suppression. This homogeneous solvation-derived SEI, dominated by interconnected LiF/B–O/B–F/Li_2_SO_4_ inorganic crystallites, establishes rapid ion-conduction channels via grain-boundary networks (Fig. 4d). In contrast, conventional electrolytes yield a porous organic-dominant SEI (e.g. ROCO_2_Li), which impedes Li^+^ diffusion (Fig. 4d). Notably, the single sulfite solvent system (1 M LiDFOB–ES) was also compared to verify the interface optimization effects of DMS and IF, and to address the concern regarding the difference without dielectric heterogeneity (∆ε = 0) (Fig. S37e–h), it confirms that the multi-solvent formulation incorporating IF favors LiDFOB decomposition over solvents, yielding a superior conductive interface, validating the suboptimal nature of single-solvent systems under the PGE strategy and underscoring the fact that modulated dielectric heterogeneity facilitates a more desirable interphase composition. Apart from the SEI, a cathode–electrolyte interphase (CEI) on the LCO cathode was also detected (Fig. S37a–d, Table S9), it reveals that IF incorporation markedly suppresses solvent decomposition (C–O content drops from 34.25% to 28.66%) and concurrently promotes LiDFOB-derived inorganic interphase formation, as evidenced by increased LiF (70.71%) and B–F/B–O species. The concomitant reduction in organic sulfur further confirms that the ES–DMS–IF system favors anion decomposition, leading to a more robust and conductive interface. Post-cycling S 2*p* analysis (Fig. S41) reveals the CEI dynamically evolves from initial organic sulfites into a highly conductive inorganic-rich structure (Li_2_S/Li_2_S_x_), which is crucial for achieving exceptional long-term cycling stability (81% capacity retention after 400 cycles at −20°C).

To evaluate the thermodynamic and kinetic advantages of optimized interfacial chemistry, we conducted *in situ* electrochemical impedance spectroscopy (EIS) at −10°C for subsequent distribution of relaxation times (DRT) analysis [53–57] (Fig. 4e and f, Figs S20 and S21). The high-frequency regimen (relaxation times 0.001–0.1 s) corresponds to interfacial impedance (R_SEI_), while the low-frequency region (0.1–10 s) reflects charge transfer resistance (R_ct_) [58]. Both components exhibit strong potential dependence, with R_ct_ dominating interfacial dynamics. In the EC–DMC system, LCO/Li cells demonstrate voltage-dependent R_SEI_ evolution. This initial reduction from 195 Ω open circuit voltage (OCV) to 61.6 Ω at 4.0 V occurs during conductive SEI formation, followed by moderate increase to 79.4 Ω upon deep delithiation (4.0–4.45 V) (Fig. 4e and g, Fig. S20a and S20e, Fig. S21a), indicating interfacial instability under high voltage. Subsequent discharge to 3.0 V triggers dramatic R_SEI_ resurgence to 193 Ω, consistent with prior observations of cyclic SEI dissolution/reformation [59–61]. This interfacial fragility contrasts sharply with sulfite-based electrolytes (ES–DMS systems) where R_SEI_ maintains lower values (<60 Ω) with minimal fluctuations post-initial formation (Fig. 4f and g, Fig. S20c and S20e, Fig. S21c), corroborating XPS-identified F-/S-/B-rich interfaces with enhanced conductivity and stability. Notably, baseline ES–DMS electrolytes reduce impedance magnitudes but fail to prevent discharge-induced SEI dissolution, whereas the ES–DMS–IF formulation achieves ultra-stable R_SEI_ (<40 Ω) throughout cycling (Fig. S20b and S20e, Fig. 21b), confirming the superior interfacial resilience of its LiF/Li_2_CO_3_ matrix reinforced by B–O/B–F/LiS_x_ species. All systems exhibit R_ct_ intensification at extreme delithiation (4.0–4.45 V) and deep lithiation (3.2–3.0 V) states, reflecting kinetically hindered Li^+^–electron coupling. Nevertheless, sulfite-based electrolytes demonstrate systematically reduced R_ct_ across all potentials, with the max R_ct_ decreased from 2811 Ω for EC–DMC to 1656 Ω for ES–DMS and 801 Ω for ES–DMS–IF. (Fig. 4g, Fig. S20f, Fig. S21), validating their homogeneous solvation structures in lowering desolvation barriers.

The homogeneous solvation feature fundamentally optimizes the entire ion transport process by synergistically linking solvation structure, desolvation dynamics and interfacial ion transfer. Unlike asymmetrical coordination configurations (Fig. 4h) that hinder desolvation kinetics and interfacial transport, the balanced coordination between anions and weakly solvating solvents in this homogeneous system reduces Li⁺ desolvation energy while promoting anion-derived inorganic interphase formation, thereby establishing low-resistance pathways for enhanced interfacial ion mobility (Fig. 4i).

**Figure 5.**
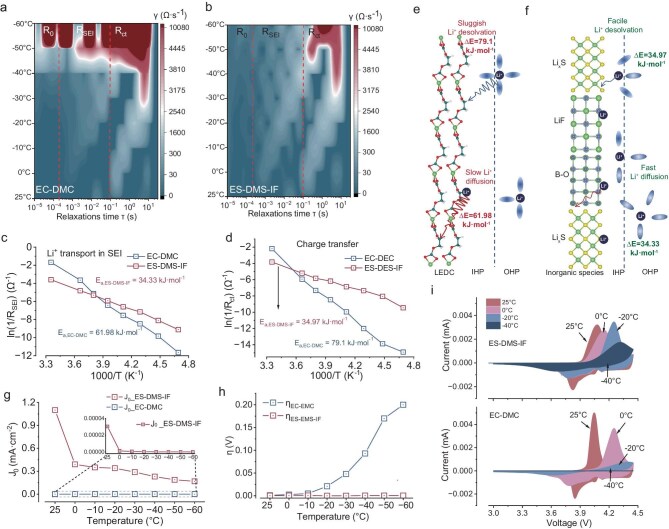
Electrochemical kinetic behaviors. (a and b) Temperature-dependent DRT mapping for the EC–DMC electrolyte (a) and ES–DMS–IF electrolyte (b) in LCO/Gr pouch cells. (c and d) Corresponding activation energies derived by Arrhenius fitting for R_SEI_ (e) and R_ct_ (f) with the above electrolytes. (e and f) Schematic of the desolvation process and Li^+^ transport within the lithium ethylene dicarbonate (LEDC)-based SEI (c) and inorganic species-based SEI (d). (g) Exchange current density of Li/Li symmetrical battery. (h) Overpotential of Li/Li symmetrical battery. (i) Temperature-dependent CV curves.

To elucidate the cryogenic kinetics of homogeneous solvation features, temperature-dependent EIS analysis (−60°C to 25°C) reveals distinct interfacial dynamics (Fig. 5a and b, Figs S22–S25, Table S4). Nyquist plot evolution shows progressive semicircle deformation. Diffusive tails vanish below 0°C, followed by semicircle truncation at −20°C and linearization at −40°C (Fig. S22). DRT analysis demonstrates temperature-dominated charge transfer behavior, with R_ct_ exhibiting higher thermal sensitivity than R_SEI_ (Fig. 5a, Fig. S23a and S24a, Fig. S25). Sulfite-based electrolytes outperform carbonates across a wide temperature range (from −60°C to 25°C), maintaining measurable resistances even at −60°C (ES–DMS–IF: R_SEI_ = 43.1 kΩ, R_ct_ = 642 kΩ vs. ES–DMS: 32.1 kΩ/401.8 kΩ) (Fig. 5b, Fig. S23b, Fig. S24b, Fig. S25), while EC–DMC systems exceed detection thresholds below −50°C (R_ct_ > 200 kΩ). The resistance hierarchy EC–DMC > ES–DMS > ES–DMS–IF persists throughout thermal cycling, with divergence amplifying at lower temperatures (ΔR_ct_ = 189% at −40°C vs. 63% at 25°C) (Figs S22–S25, Table S4), confirming homogeneous solvation’s critical role in low-temperature interfacial stabilization. Based on the temperature-dependent DRT data, the temperature-governed Li^+^ transport kinetics were quantified via the Arrhenius relationship [10]:


(1) \begin{equation*}
ln\frac{1}{{\textit{Resistance}}} = - \frac{{{E}_a}}{{RT}} + C,
\end{equation*}

where *Resistance* denotes R_SEI_ and R_ct_, *E_a_* represents the activation energy for Li^+^ diffusion (interfacial) or charge transfer, *R* is the gas constant, *T* is absolute temperature, and *C* is a pre-exponential factor. In this equation, ln (1/*Resistance*) and 1/T have a linear relationship.

Arrhenius analysis of temperature-dependent resistance (within the range –40°C to 25°C) reveals critical activation energy reduction in sulfite-based electrolytes (Fig. 5c and d, Fig. S26). The homogeneous solvation feature achieves substantially lower activation energies (*E_a_,_SEI_* = 34.33 kJ·mol^⁻¹^, *E_a_,_ct_* = 34.97 kJ·mol^−1^) compared to carbonate systems (*E_a_,_SEI_* = 61.98 kJ·mol^−1^, *E_a_,_ct_* = 79.1 kJ·mol^−1^) (Fig. 5c and d, Fig. S26). This 45%–56% energy barrier reduction directly correlates with LiF/B–O/Li_x_S interfacial components that simultaneously facilitate desolvation (desolvation energy reduced from 79.1 kJ·mol^−1^ to 34.97 kJ·mol^−1^, Fig. 5e and f) and SEI ion transport (transport energy barrier decreased from 61.98 kJ·mol^−1^ to 34.33 kJ·mol^−1^, Fig. 5e and f), confirming the synergistic optimization of solvation thermodynamics and interfacial kinetics in cryogenic operation.

Tafel analysis of Li/Li symmetrical cells reveals superior interfacial kinetics in sulfite-based electrolytes (Fig. 5g and h, Figs S27 and S28). The homogeneous solvation feature achieves cryogenically stable exchange current densities (j_0_ = 0.168 mA·cm^−2^ at −60°C for ES–DMS–IF vs. 1.2 × 10^−10^ mA·cm^−2^ for EC–DMC), demonstrating a 7–9 orders of magnitude enhancement that correlates with suppressed side reactions. Overpotential (η) evolution further confirms interfacial stability: ES–DMS–IF maintains minimal polarization (η = 4.3 × 10^−4^ V at −60°C) versus EC–DMC’s dramatic η surge (0.2 V), directly linking homogeneous solvation to optimized desolvation kinetics and reduced activation barriers under cryogenic conditions. Variable-temperature cyclic voltammetry (CV) analysis of LCO/Li cells demonstrates exceptional electrochemical reversibility in sulfite-based electrolytes (Fig. 5i, Fig. S29). While carbonate systems exhibit complete redox peak disappearance below −20°C and linear CV profiles at −40°C, the homogeneous solvation feature maintains well-defined redox couples with minimal potential shift (ΔE < 0.12 V from 25°C to −40°C). Notably, ES–DMS–IF achieves 89% room-temperature capacity retention at −40°C with a polarization increase of <15 mV/K, confirming stabilized desolvation kinetics and interfacial charge transfer under cryogenic conditions.

### Cycling behaviors under low temperature and safety performance

**Figure 6.**
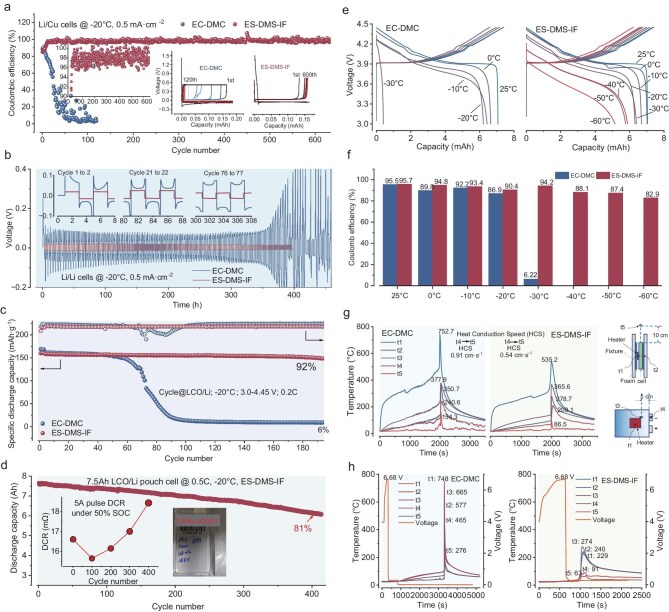
Low-temperature cycling performance and abuse test with the designed electrolytes and common electrolyte. (a) CE of Li/Cu battery. (b) Cycling performance of Li/Li symmetrical battery. (c) −20°C cycling performance of LCO/Li coin cell. (d) −20°C cycling performance of 450 Wh·kg^−1^ LCO/Li pouch cell, with 5 A pulse DCR inserted. (e) The temperature-dependent charge and discharge profiles of LCO/Li pouch cell within the range of −60°C to 25°C. (f) The corresponding CE of temperature-dependent cycling profile. (g) Heating test of 450 Wh·kg^−1^ LCO/Li pouch cell. (h) Overcharge test of 450 Wh·kg^−1^ LCO/Li pouch cell.

Cryogenic cycling (−20°C) of Li/Cu and Li/Li symmetrical cells validates the homogeneous solvation feature’s performance superiority (Fig. 6a and b). Carbonate-based electrolytes exhibit rapid failure (20 cycles, polarization surge to 0.08 V), while sulfite systems achieve unprecedented stability: Li/Cu cells maintain an average 97.8% coulombic efficiency (CE) over 600 cycles at −20°C (Fig. 6a, Fig. S30) and an average 98.4% over 200 cycles at 25°C under 0.5 mA·cm⁻^2^ (Fig. S40), even reaching an average CE of 48.4% under much higher current density of 1.0 mA·cm⁻^2^ (Fig. S42). Li/Li cells sustain ultralow overpotential (<25 mV) using ES–DMS–IF electrolyte with a current density of 0.5 mA·cm⁻^2^ and a deposition capacity of 1 mAh·cm⁻^2^ (Fig. 6b). Notably, ES–DMS–IF outperforms ES–DMS with 35% lower polarization (0.013 vs. 0.020 V at 2000 h) under a current density of 0.05 mA·cm⁻^2^ and a deposition capacity of 1 mAh·cm⁻^2^ (Fig. S31), directly correlating with its inorganic-rich interphase that enables efficient Li deposition (CE: 99.92% ± 0.05% vs. 99.81% ± 0.12% in ES–DMS). Moreover, the overpotentials of ES–DMS–IF electrolyte show slight changes despite the 10-fold difference in current density, reflecting the less current density-related polarization behavior. These metrics establish homogeneous solvation as a viable pathway toward cryogenic lithium metal batteries.

Cryogenic cycling (−20°C, 0.2 C) of LCO/Li cells further demonstrate hierarchical performance (Fig. 6c, Figs S32 and S33); EC–DMC fails abruptly at 60 cycles (polarization surge > 0.5 V), while ES–DMS delivers intermediate stability (69% capacity retention at 200 cycles) and ES–DMS–IF excels with 92% retention and minimal polarization growth (Δη < 15 mV/cycle). Notably, we also compared with the single sulfite solvent systems (1 M LiDFOB–ES) to valid the optimal formula (Fig. S38); it exhibited poor cryogenic performance due to high melting point and insufficient interphase stability, while the single-solvent may eliminate the dielectric heterogeneity. Commercial validation using 7.5 Ah pouch cells reveals practical viability under −20°C and 0.5 C. ES–DMS–IF achieves 81% capacity retention over 400 cycles (Fig. 6d) with ultralow DCR increase (16.6 to 18.8 mΩ, Δ ∼ 13%, inset in Fig. 6d), outperforming conventional electrolytes. This hierarchy (EC–DMC < ES–DMS < ES–DMS–IF) directly correlates with enhanced interfacial stability from anion-coordinated solvation structures, further confirming their cold-climate operational superiority. Temperature-dependent cycling of 7.5 Ah LCO/Li pouch cells again reveals hierarchical electrolyte performance (Fig. 6e and f, Figs S34 and S35, Tables S5 and S6): EC–DMC systems fail below −30°C (0% discharge capacity), while ES–DMS maintains 30.7% capacity retention at −60°C. The ES–DMS–IF electrolyte demonstrates cryogenic superiority with 91%/94.2% capacity retention/CE at −30°C (6.4 Ah discharge) and 73.3%/82.9% retention/efficiency at −60°C (5.16 Ah), outperforming ES–DMS by 2.4 times in low-temperature operational stability. This performance hierarchy (EC–DMC < ES–DMS < ES–DMS–IF) directly correlates with interfacial ion transport optimization through anion-coordinated solvation networks, enabling functional charge transfer even under extreme polarization (Δη < 0.6 V at −60°C). This breakthrough establishes homogeneous solvation design as a viable solution for ultralow-temperature lithium battery operation, with performance comparable to state-of-the-art cryogenic systems [2,16,62–64]. Furthermore, accelerated aging tests of LCO/Li pouch cells (60°C/7 days) confirm the outstanding bulk stability of the ES–DMS–IF electrolyte, as ^1^H nuclear magnetic resonance (NMR) spectra reveal no detectable changes in chemical shifts or new degradation peaks (Fig. S42). This demonstrates exceptional chemical and thermal integrity under harsh conditions, corroborating the robustness of the formulated electrolyte.

Safety evaluation through thermal/electrical abuse tests confirms the superior stability of sulfite-based electrolytes. In heating tests (Fig. 6g), ES–DMS–IF cells exhibit 29% lower maximum temperature (535°C vs. 752.7°C in EC–DMC) and 41% reduced heat conduction speed (0.54 cm·s^−1^). Overcharge testing (Fig. 6h) reveals critical thermal runaway mitigation: EC-DMC cells reach catastrophic temperatures (>700°C) within 721 s, while ES–DMS–IF systems maintain thermal stability below 274°C throughout 3000 s testing. This 62%–89% temperature reduction across five monitoring points (t1–t5) directly correlates with the electrolyte’s self-terminating decomposition mechanism, demonstrating practical viability for high-safety battery applications.

## CONCLUSION

In summary, this study establishes a transformative PGE paradigm that resolves the fundamental solvation dichotomy crippling cryogenic lithium-ion batteries by strategically compressing dielectric heterogeneity (Δε) through atomic-scale sulfur substitution in carbonate frameworks, achieving balanced Li⁺ coordination among high-/low-polarity solvents and anions. The resultant homogenized solvation feature decouples three critical limitations of conventional electrolytes: (i) thermodynamic rebalancing via weakened preferential coordination with strong-polar solvents reduces desolvation energy barriers by 45%–56% (34.97 vs. 79.1 kJ·mol^−1^ in carbonates); (ii) kinetic synergy between anion participation and weak-polar solvents drives inorganic-rich interphase formation (LiF/B–O/Li_x_S content > 84%), enabling ultralow interfacial resistance (R_SEI_ < 40 Ω) and dendrite-free Li deposition (CE: 99.92% at −20°C); (iii) self-optimized solvation microdomains ensure superior ionic conductivity (1 mS·cm⁻^1^ at –80°C) and phase stability (liquid down to −110°C). Validated in practical 7.5 Ah pouch cells, the PGE-driven sulfite electrolyte achieves unprecedented cryogenic performance: 81% capacity retention over 400 cycles at −20°C and 73.3% capacity delivery at −60°C with outperforming safety performance. Beyond empirical electrolyte design, this work pioneers a universal ‘polarity gradient–solvation homogeneity–interfacial kinetics’ framework that overcomes the fundamental trade-off between ionic mobility and desolvation barriers, providing a molecular blueprint for next-generation cryogenic energy storage systems.

## METHODS

### Electrolyte preparation and battery fabrication

The baseline EC–DMC electrolyte contained 1 M LiPF_6_ in EC:DMC (3:7 by vol%). Test electrolytes consisted of 1 M LiDFOB in sulfite/carboxylate solvent mixtures: ES–DMS (ES:DMS = 3:7) and ES–DMS–IF (ES:DMS:IF = 2:4.5:3.5 by vol%). All preparations were conducted in an argon-filled glovebox (H_2_O, O_2_ < 0.01 ppm). A standard 7.5 Ah LCO/Li pouch cell (electrochemical window: 3–4.45 V) was fabricated by injecting 11.25 g of electrolyte, followed by a sequence of initial sealing, formation, gas venting and final secondary sealing. LCO/Li and Li/Li coin cells were assembled using a LiCoO_2_ cathode (specific surface area: 1.88 m^2^·g⁻^1^, measured via nitrogen adsorption–desorption) and a lithium metal anode.

### Electrolyte characteristic

Electrolyte conductivity was measured using a conductivity meter within a temperature-controlled cold bath (ethanol/liquid nitrogen mixture). FTIR spectra (KBr tablet, 4000–600 cm⁻^1^ range) and Raman spectra (532 nm laser, 2 mW power) were collected. Samples were equilibrated at the target temperature using the same cooling device prior to spectroscopic analysis.

### Electrochemical measurements

Galvanostatic charge/discharge tests for coin and pouch cells were performed using battery test systems. Cells were temperature-controlled in an environmental chamber during testing. EIS was conducted from 0.01 Hz to 100 kHz with a 10 mV amplitude. Cyclic voltammetry (3.0–4.45 V vs. Li/Li⁺, 0.2 mV/s) and Tafel tests (in Li/Li symmetrical cells, −0.2 to 0.2 V, 0.5 mV/s) were carried out on an electrochemical workstation.

### Material characterizations

The interfacial composition of LiCoO_2_ cathodes was analyzed by XPS with a vacuum transfer vessel. Spectral data were processed using commercial software, and atomic concentrations were determined using relative sensitivity factors. Differential scanning calorimetry (DSC), viscosity and conductivity measurements were performed using respective specialized instruments.

### Computational methods

DFT calculations were performed with Gaussian 16 [65] at the B3LYP/6-311G level [66], including GD3BJ dispersion correction, for geometry optimization, vibrational frequency and bond energy analysis. Molecular van der Waals volumes were calculated using Multiwfn [67,68]. Classical molecular dynamics (cMD) simulations employed GROMACS 2024.6 [69]. Electrolyte systems (carbonate: 5 LiPF_6_/22 EC/40 DMC; sulfite: 5 LiDFOB/13 ES/25 DMS/15 IF) were packed at an initial density of 1.2 g/cm^3^ using GAFF force fields [70,71] with 0.8 charge scaling for ions. Simulations used a 1 fs timestep under periodic boundary conditions. Systems underwent energy minimization, 5 ns NPT equilibration at 25°C, and 50 ns NVT production runs. For −60°C simulations, an 850 ps annealing step preceded equilibration. Trajectories were analyzed for radial distribution functions and mean square displacement.

Detailed method information can be found in the online Supplementary data.

## Supplementary Material

nwaf543_Supplemental_File
