# Supplementary material for: Decoding polarity gradient enabled ultra-high lithium ion conduction
Source: Natl Sci Rev. 2025 Nov 29;13(3):nwaf543. doi: 10.1093/nsr/nwaf543 (PMC12887302; doi:10.1093/nsr/nwaf543)
Supplement: nwaf543_Supplemental_File [file nwaf543_supplemental_file.pdf]

Decoding Polarity Gradient Enabled Ultra-high Lithium Ion Conduction

Yuqing Chen[1,2#], Aiping Wang[3#], Yun Zhao[4#], Wei Wang[1], Robert Dominko[3], Peitao Xiao[5], Peng Gao[1], Yan Duan[1], Baohua Li[4], Xiangming He[6], Jilei Liu[1*]

[1]*College of Materials Science and Engineering, Hunan Joint International Laboratory of Advanced Materials and Technology of Clean Energy, Hunan Province Key Laboratory for Advanced Carbon Materials and Applied Technology, Hunan University, Changsha 410082, People's Republic of China*

[2]*Zhejiang Collaborative Innovation Center for Full-Process Monitoring and Green Governance of Emerging Contaminants , Interdisciplinary Research Academy, Zhejiang Shuren University, Hangzhou, 310021, China*

[3]*Department of materials chemistry, National Institute of Chemistry, Haidrihova 19, 1000 Ljubljana, Slovenia*

[4]*Shenzhen Key Laboratory on Power Battery Safety and Shenzhen Geim Graphene Center, Tsinghua Shenzhen International Graduate School (SIGS), Shenzhen 518055, China*

[5]*College of Aerospace Science and Engineering, National University of Defense Technology, Changsha 410073, China*

[6]*Institute of Nuclear and New Energy Technology, Tsinghua University, Beijing 100084, China*

The definition of the abbreviated solvent molecules.

Category	Abbreviation	Full name	CAS No.	Molecular structural
Cyclic solvent molecules	EC	Ethylene carbonate	96-49-1	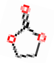
	PC	Propylene carbonate	108-32-7	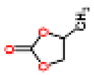
	FEC	Fluoroethylene carbonate	114435-02-8	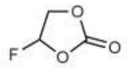
	VC	Vinylene carbonate	872-36-6	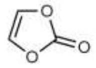

	GBL	Gamma butyrolactone	96-48-0	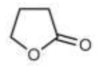
	DOL	1,3-Dioxolane	646-06-0	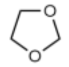
	THF	Tetrahydrofuran	109-99-9	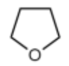
	SL	Sulfolane	126-33-0	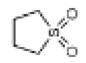
	ES	Ethylene sulfite	3741-38-6	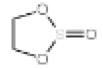
	DTD	1,3,2-Dioxathiolane 2,2-dioxid	1072-53-3	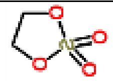
Linear carboxylic esters	EA	Ethyl acetate	141-78-6	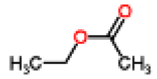
	IF	Isobutyl formate	542-55-2	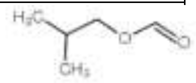
	EB	Ethyl Butanoate	105-54-4	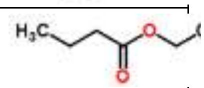
	EHFB	Ethyl heptafluorobutyrate	356-27-4	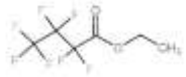
	DMFA	Methyl difluoroacetate	433-53-4	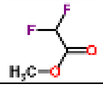
	DMFSA	Methyl 2,2-difluoro-2-(fluorosulfonyl)acetate	680-15-9	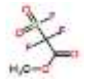
Linear carbonates	DMC	Dimethyl carbonate	616-38-6	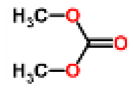
	DEC	Diethyl carbonate	105-58-8	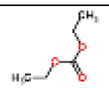
	EMC	Ethyl methyl carbonate	623-53-0	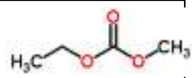
ether	DME	1,2-Dimethoxyethane	143585-58-4	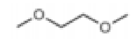
	D2	1,1,2,2-Tetrafluoroethyl-2,2,3,3-tetrafluoropropylether	16627-68-2	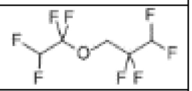
Phosphate/ borates	TMP	Trimethyl phosphate	512-56-1	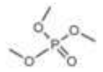
	TMB	Trimethyl borate	121-43-7	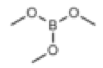
Linear sulfate/ sulfone	DES	Diethylsulfit	623-81-4	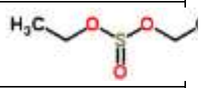
	DMS	Dimethyl sulfite	616-42-2	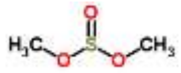

	EMS	Ethyl methyl sulfone	594-43-4	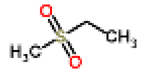
Nitrile	AN	Acetonitrile	75-05-8	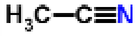
	VN	Valeronitrile	110-59-8	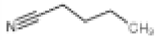
	SN	Succinonitrile	110-61-2	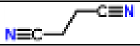
	ADN	Adiponitrile	111-69-3	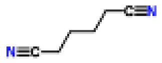
	FAN	Fluoroacetonitrile	503-20-8	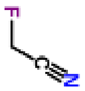

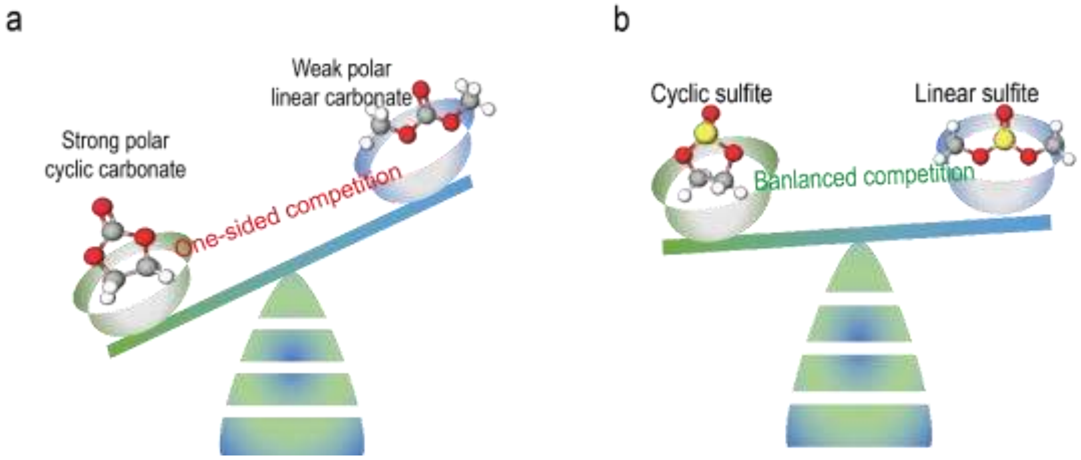

Fig S1. The coordination competition situation of mix solvents in the traditional electrolytes (a) and our proposed electrolytes (b).

Impact of Dielectric Heterogeneity in Mixed-Solvent Electrolytes for Lithium-Ion Batteries. Significant dielectric mismatch in mixed-solvent electrolytes (i.e., dielectric heterogeneity) profoundly influences lithium-ion battery performance through multiple mechanisms:

1. Solvation Structure Heterogeneity

High-dielectric solvents (e.g., EC, γ-BL) preferentially solvate Li^+ ions due to strong dipole interactions, forming localized Li^+-rich solvation clusters. Low-dielectric solvents (e.g., DMC, DME) exhibit weaker solvation power, leading to uneven Li^+ distribution and incomplete salt dissociation. This heterogeneity disrupts homogeneous Li^+ flux, aggravating concentration polarization and dendritic Li deposition[1].

2. Ion Transport Dynamics

Dielectric disparity induces spatially nonuniform ionic conductivity[2-4]. High-dielectric domains facilitate ion dissociation but may trap Li^+ via excessive coordination, while low-dielectric regions promote ion pairing (e.g., [Li^+-PF_6^-]), reducing effective charge carriers. This imbalance lowers bulk ionic conductivity and increases charge-transfer resistance, particularly at low temperatures.

3. Interfacial Stability

Competing solvation tendencies destabilize the electrode-electrolyte interface[5-7]. High-dielectric solvents dominate the Li^+ solvation sheath, driving preferential decomposition at the anode (e.g., EC → polymeric SEI). Meanwhile, low-dielectric components may aggregate at

interfaces, forming inhomogeneous SEI/CEI with weak mechanical integrity, accelerating parasitic reactions and capacity fade.

4. Electrochemical Performance Trade-offs

While high-dielectric solvents enhance salt dissociation, excessive dielectric heterogeneity exacerbates Li^+ desolvation barriers at the anode interface due to mismatched solvation energies[1,8]. This compromises rate capability and cycle life. Notably, such effects are amplified under extreme conditions (e.g., high-rate charging, subzero temperatures).

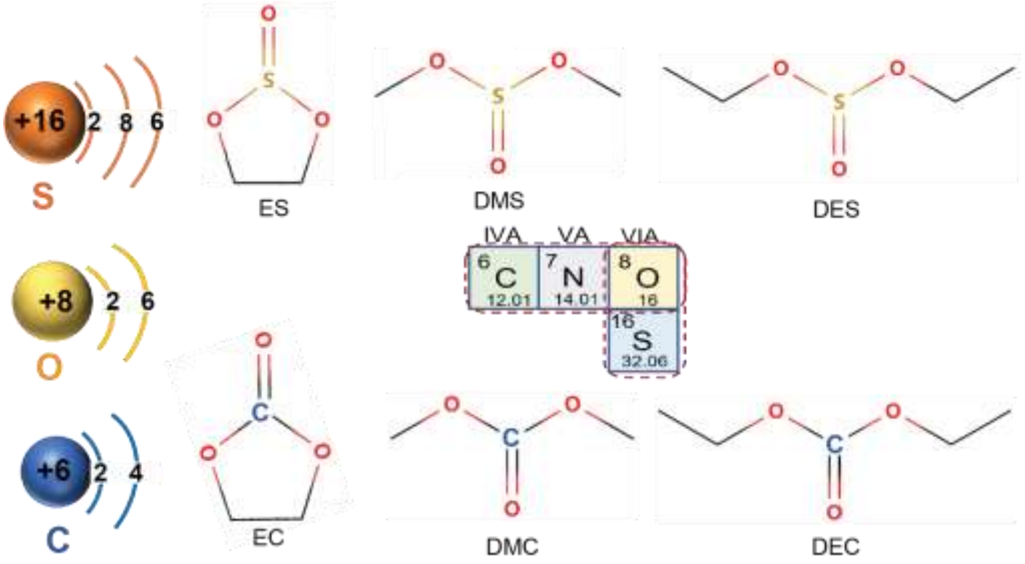

Fig S2. Comparison of typical carbonate solvents and sulfite solvent structures.

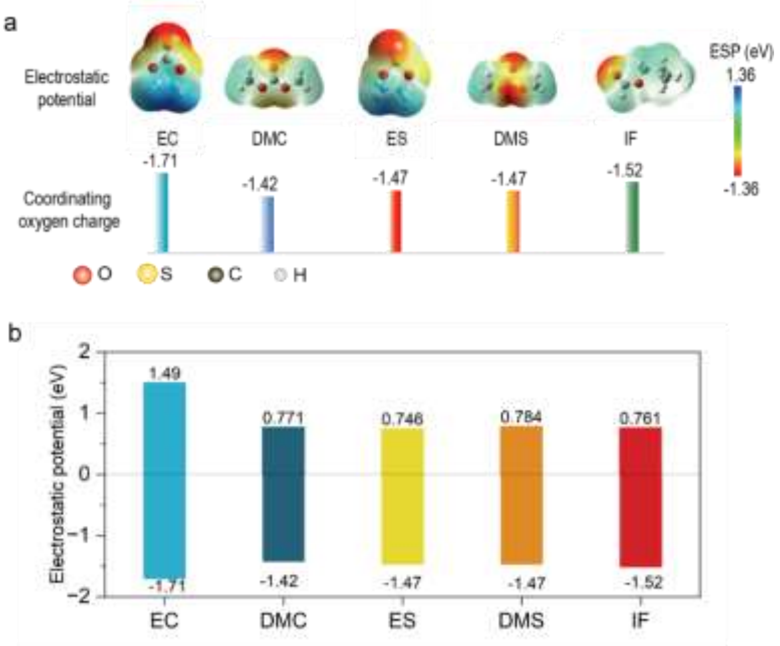

Fig S3. Typical electrostatic potential of carbonate solvents, sulfite solvents and isobutyl formate. (a) electrostatic distribution of the above solvents, (b) the electrostatic potential extremes.

In the carbonate group, the electronegativity and electrostatic potential of EC and DMC are very different, which easily forms a one-sided coordination trend. In the sulfite group, the

electronegativity and IF extreme value difference between DMS and ES are very small, which further proves that the coordination ability of ES, DMS, and IF is homogenized.

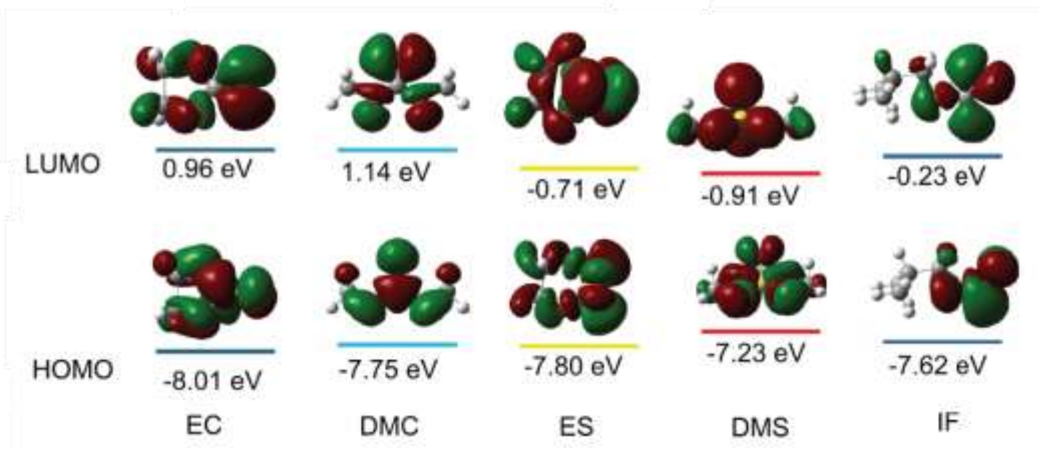

Fig S4. Highest occupied molecular orbital (HOMO) and lowest unoccupied molecular orbital (LUMO) energy level of carbonate solvents, sulfite solvents and isobutyl formate.

Sulfite has higher reduction activity and is easily decomposed at the negative electrode to form sulfur-containing interface components.

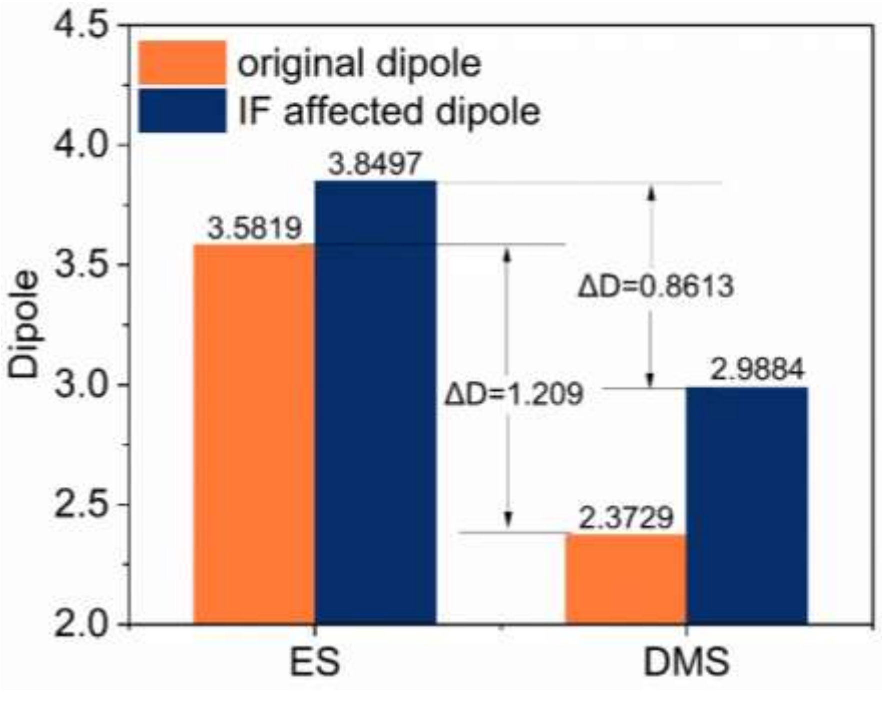

Fig S5. Effect of Isobutyl Formate Electrolyte on the Polarity of Sulfite Solvents ES and DMS.

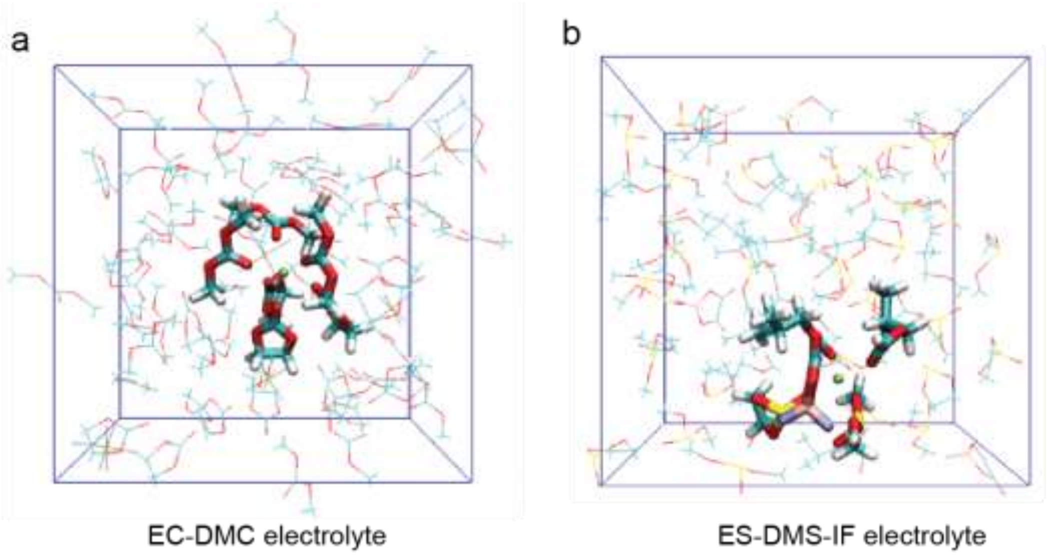

Fig S6 Snapshots of EC-DEC (a) and ES-DMS-IF electrolytes (b) obtained by MD simulation at 298 K. The Li+ and their primary coordinated shells (within 2.6 Å of Li^+ ions) are presented by ball and stick models, while the wireframes stand for free solvents.

Calculation Method

The DFT calculations were carried out by Gaussian 16[9]. All the optimization, frequency calculation and bond energy scanning were conducted at the B3LYP/6-311G level.[10] Energy dispersion was corrected by GD3BJ method.[11] The van der Waals volume of molecules were extracted with Multiwfn[12,13].

Classical molecular dynamics (cMD) simulations were performed using the GRMOACS 2024.6 package under periodic boundary conditions.[14] The molecules were packed into a cubic box by PACKMOL[15] with a tolerance of 2.5 Å and an initial density of 1.2 g/cm^3. The carbonate-based electrolyte is formulated with 5 LiPF6, 22 EC and 40 DMC molecules. The sulfite-based electrolyte was formulated with 5 LiDFOB, 13 ES, 25 DMS and 15 IF molecules. Both temperatures at 25 and -60 ℃ were modelled. The general amber force field (GAFF)[16] were generated with Sobtop[17] and the charges of ions were scaled by a factor of 0.8. A timestep of 1 fs was used for all molecular calculations. The dispersion correction was used for van der Waals interaction description. Atomic electrostatic interaction was calculated by particle mesh Ewald (PME) method. A cut-off less than half the box size was used to avoid self-interactions. The LINCS (Linear Constrain Solver) algorithm was used to impose constraints on the hydrogen bond. cMD simulations started from energy minimization using conjugate gradient minimization scheme with a step size of 0.01 nm. A maximum force of 50 kJ mol^{-1} nm^{-1} was reached. A 5 ns

equilibration was conducted under the NPT ensemble. The V-rescale and Berendsen methods were used for temperature and pressure coupling. The systems were equilibrated to 25 ℃ and 1.01325 bar. The densities of carbonated-based and sulfite-based electrolytes reached 1.267 g cm^{-3} and 1.269 g cm^{-3}, respectively, which agree well with experimental results. The equilibrated systems were used for50 ns production run under NVT. As for the -60 ℃ cMD simulations, additional annealing was performed from 25 to -60 ℃ in 850 ps under the NVT ensemble and followed by a 5 ns equilibration (NPT) before production run. The densities of carbonated-based and sulfite-based electrolytes reached 1.328 g cm^{-3} and 1.307 g cm^{-3}, respectively, as expected at lower temperatures. Post analysis was performed based on production runs. Structural visualization was realized within VMD.[18]

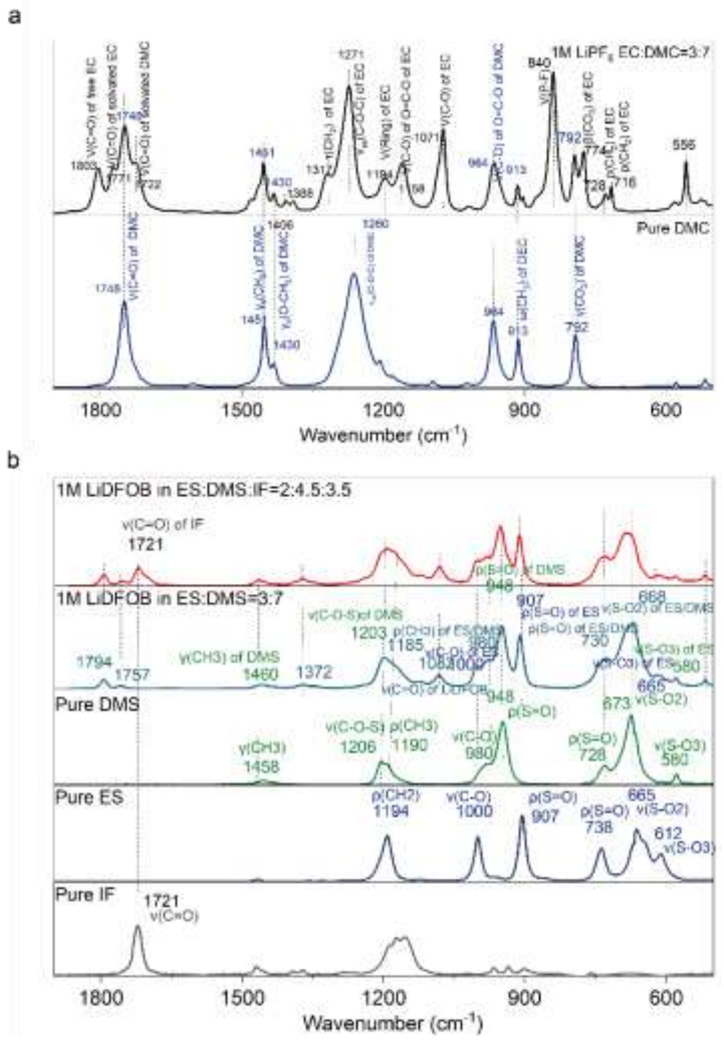

Fig S7. Comparative analysis of the infrared spectra of electrolytes and the corresponding pure solvents. (a) Carbonate electrolyte; (b) Sulfite electrolyte.

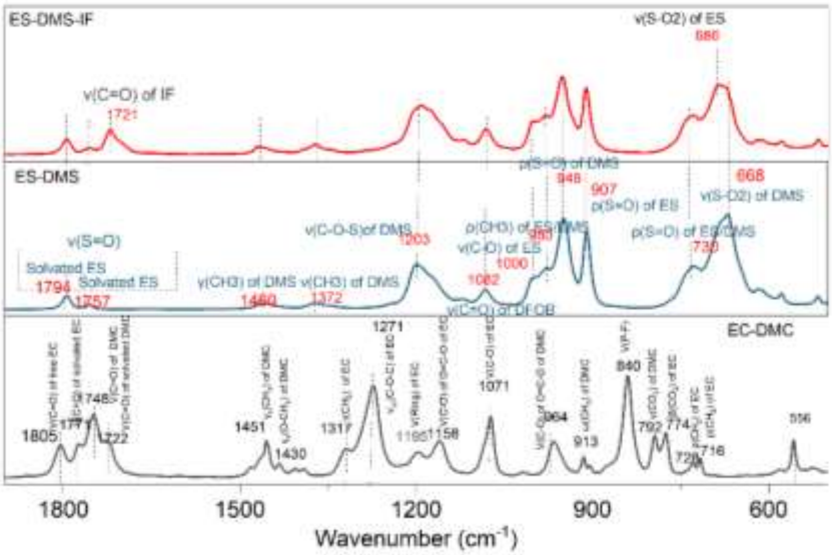

Fig S8. Results of infrared spectrum peak position analysis of three electrolytes.

The above three electrolytes were analyzed by infrared and Raman spectra. Fig S6-7 show the analysis results of infrared spectra of EC-DMC, ES-DMS and ES-DMS-IF. In the EC-DEC electrolyte system, the stretching vibration peak of carbonyl C=O appears in the range of 1700~1810 cm^{-1}, the bending vibration peak of CH_2/CH_3 appears at around 1400 cm^{-1}, the stretching vibration peak of carbonate O-C=O appears at around 1300 cm^{-1}, and the P-F stretching vibration peak appears at 850 cm^{-1}. In order to further attribute and identify each specific peak position, we compared the infrared spectra of the electrolyte and the pure solvents that make up this electrolyte (Fig S7). For example, in the C=O band, the vibration peak of the electrolyte at 1748 cm^{-1} is highly consistent with the C=O stretching vibration peak of pure DMC, so this peak is attributed to the C=O stretching vibration of linear carbonate DMC (free DMC vibration), while the shoulder peak at 1722 cm^{-1} at the low-frequency position on its right side belongs to the C=O stretching vibration of DMC coordinated with Li^+, and the 1802 cm^{-1} and 1771 cm^{-1} in the high-frequency band are respectively attributed to the C=O stretching vibration of free EC and the C-O stretching vibration peak of EC coordinated with Li^+. In the C=O band range, the infrared signal of free DMC solvent is the strongest, and the solvated EC is dominant, so in the EC-DMC electrolyte, the strong polar EC solvent is mainly involved in the solvation structure.

For sulfite electrolyte, by comparing the main peaks of each pure solvent, it is found that the infrared signal of sulfite electrolyte mainly comes from S=O stretching vibration. The vibration signals of cyclic sulfite ES and linear sulfite DMS are concentrated in the range of 600-1220cm^{-1}. The main peaks of strong infrared signals of ES and DMS are attributed to the swing vibration of S=O, which are located at 907cm^{-1} and 948cm^{-1}, respectively. Therefore, infrared vibrations of these two wave numbers appear simultaneously in the infrared spectrum of sulfite electrolyte. In addition, the O-S-O stretching vibration of ES and DMS are located at 665 cm^{-1} and 673 cm^{-1}, respectively, and the stretching vibration of O=S-O_2 is located at 612 cm^{-1} and 580 cm^{-1}, respectively, so the superposition of these vibration signals appears in sulfite electrolyte. The shoulder peaks at 1203 cm^{-1} and 1185 cm^{-1} correspond to the C-O-S stretching vibration of DMS and the CH3 swing vibration of ES/DMS, respectively. It is worth noting that the C=O stretching vibration peak of the lithium salt LiDFOB appears at 1082 cm^{-1}, and two weak new peaks appear

at 1794 and 1757 cm^{-1}, which are also attributed to the vibration signal of the lithium salt LiDFOB. In addition, compared with the ES-DMS electrolyte, the ES-DMS-IF electrolyte has a new peak at 1721 cm^{-1}, which is highly consistent with the C=O stretching vibration of the pure solvent of isobutyl formate, so this peak is attributed to the C=O stretching vibration of isobutyl formate. In general, compared with the dominant coordination of EC to Li^+ in carbonate electrolytes, the coordination peak signals of ES and DMS in sulfite electrolytes are almost equal, which further verifies the homogeneous solvation structure of ES and DMS, and provides experimental support for the above theoretical basis. The above infrared peak decomposition results (peak position attribution) are finally presented in Fig S7-8 and Table S1

Table S1. Attribution of peak positions of infrared spectra of three electrolytes

No	EC-DMC		Sulfite electrolyte		
			ES-DMS	ES-DMS-IF	
	Wavenumber (cm^{-1})	Peak assignment	Wavenumber (cm^{-1})	Wavenumber (cm^{-1})	Peak assignment
1	1805	ν(C=O) of free EC[19-21]	1794	1794	Solvated ES[22,23]
2	1771	ν(C=O) of solvated EC[19-21]	1757	1757	Solvated ES[22,23]
3	1748	ν(C=O) of free DMC[19-21]		1721	ν(C=O) of IF[24]
4	1722	ν(C=O) of solvated DMC[19-21]	1460	1460	γ(CH3) of DMS[22,23]
5	1451	γ_s(CH3) of DMC[25]	1372	1372	ν(CH3) of DMS[22,23]
6	1430	γ_s(O-CH3) of DMC[25]	1203	1203	ν(C-O-S)of DMS[22,23]
7	1317	τ(CH2) of EC[25]	1082	1082	ν(C=O) of DFOB[-26-29]
8	1271	ν_{as}(C-O-C) of EC[25]	1000	1000	ν(C-O) of ES[22,23]
9	1195	ν(Ring) of EC[25]	980	980	ρ(CH3) of ES/DMS[22,23]
10	1158	ν(C-O) of O=C-O of EC[25]	948	948	ρ(S=O) of DMS[22,23]
11	1071	ν(C-O) of EC[25]	907	907	ρ(S=O) of ES[22,23]
12	964	ν(C-O) of O=C-O of DMC[25]	730	730	ρ(S=O) of ES/DMS[22,23]
13	913	ω(CH3) of DMC[30]		686	ν(S-O2) of ES[22,23]
14	840	ν(P-F)[31]	668	668	ν(S-O2) of DMS[22,23]
15	792	γ(CO3) of DMC[25]			
16	774	β(CO3) of EC[25]			
17	728	ρ(CH2) of EC[25]			
18	716	ρ(CH2) of EC[25]			

*注: ν_s: Symmetrical stretching vibration ν_{as}: Asymmetric stretching vibration
β: In-plane bending vibration   γ: Out-of-plane bending vibration
δ_s: Symmetrical deformation vibration δ_{as}: Asymmetric deformation vibration
δ: Shear vibration   ρ: Swing Vibration
ω: In-plane rocking vibration   τ: Torsional vibration

In order to further verify the rationality and accuracy of the above solvation structure analysis, we conducted Raman spectroscopy tests on the above electrolytes, and used a similar spectral analysis method to the above infrared spectroscopy to compare and analyze the Raman spectra of

the electrolytes with those of the corresponding pure solvents (Fig S9). For the EC-DMC electrolyte, the Raman signals of EC appeared at ~730 cm^{-1} and ~900 cm^{-1}, corresponding to the symmetric deformation vibration of the EC ring and the symmetric stretching vibration of C-O, respectively; the $PF6^{-}$ stretching vibration signal appeared at 745 cm^{-1}; and a strong peak appeared at 920 cm^{-1}, which was highly consistent with the C-O symmetric stretching vibration peak of the pure DMC solvent. Therefore, from the Raman analysis results, EC still dominates the solvation structure of lithium ions in the EC-DMC electrolyte.

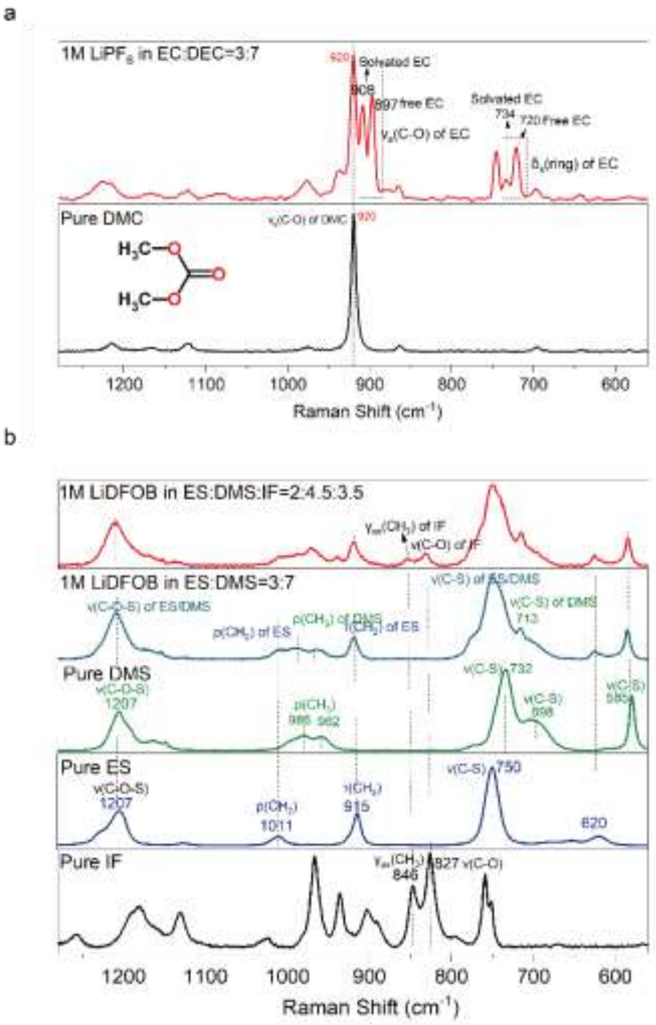

Fig S9. Comparative analysis of the Raman spectra of electrolytes and the corresponding pure solvents. (a) Carbonate electrolyte; (b) Sulfite electrolyte.

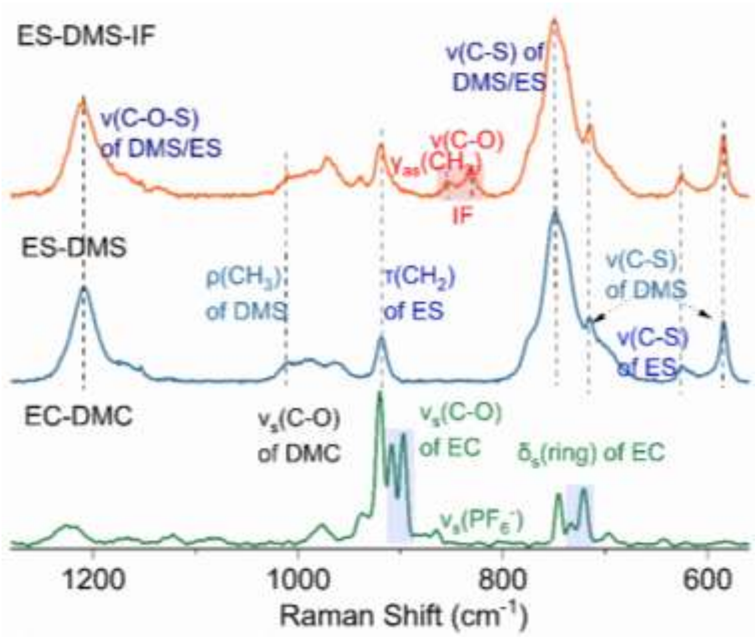

Fig S10. Results of peak position analysis of Raman spectra of three electrolytes.

For sulfite electrolyte, the Raman peak positions of ES and DMS are relatively equal. For example, the C-O-S stretching vibration peaks of pure solvent DMS and ES appear at 1207 cm^{-1} wave number, so the Raman peak at 1207 cm^{-1} in sulfite electrolyte is the superposition of ES and DMS Raman signals. Similarly, the Raman main peak at 750 cm^{-1} wave number is the superposition of ES and DMS C-S stretching vibrations, and the range of 1020-915 cm-1 is also the superposition of ES and DMS CH_2/CH_3 swing vibrations. Therefore, in sulfite electrolyte, ES and DMS signals are almost equal, which once again confirms that the solvation effects of ES and DMS on Li^+ are equal, which provides experimental basis for the homogeneous solvation structure theory. In addition, compared with ES-DMS, ES-DMS-IF electrolyte has new peaks at 846 cm^{-1} and 827 cm^{-1}, which correspond to the asymmetric out-of-plane bending vibration and C-O stretching vibration of CH_3 in pure isobutyl formate IF solvent, respectively. Therefore, the Raman spectrum analysis is consistent with the infrared spectrum analysis results. The above Raman peak spectrum analysis results are presented in Fig S9-10 and Table S2.

Table S2. Attribution of peak positions of Raman spectra of three electrolytes

No.	EC-DMC		Sulfite electrolyte		
	Wavenumber (cm^{-1})	Peak assignment	ES-DMS Wavenumber (cm^{-1})	ES-DMS-IF Wavenumber (cm^{-1})	Peak assignment Wavenumber (cm^{-1})
1	—	—	585	585	ν(C-S) of DMS[32,33]
			625	625	ν(C-S) of ES[32,33]
2	720	δ_s(ring) of free EC[34] γ(ring) of C=O of EC[35]	716	716	ν(C-S) of DMS[32,33]
3	734	δ_s(ring) of solvated EC[34]	748		ν(C-S) of DMS/ES[32,33]
4	745	$\nu_s(PF_6^-)$ of $LiPF_6$[35]	—	831	ν(C-O) of IF
5	897	ν_s(C-O) of free EC[34]	—	855	ν(C-O) of IF

		ring breath of free EC[35]			
6	908	ν_s(C-O) of solvated EC[34] ring breath of solvated EC[35]	920	920	τ(CH2) of ES[32,33]
7	920	ν_s(C-O) of DMC	962	938	ρ(CH3) of DMS[32,33]
8			986	971	
9			1011	1011	ρ(CH2) of ES[32,33]
10			1209	1211	ν(C-O-S) of DMS/ES[32,33]

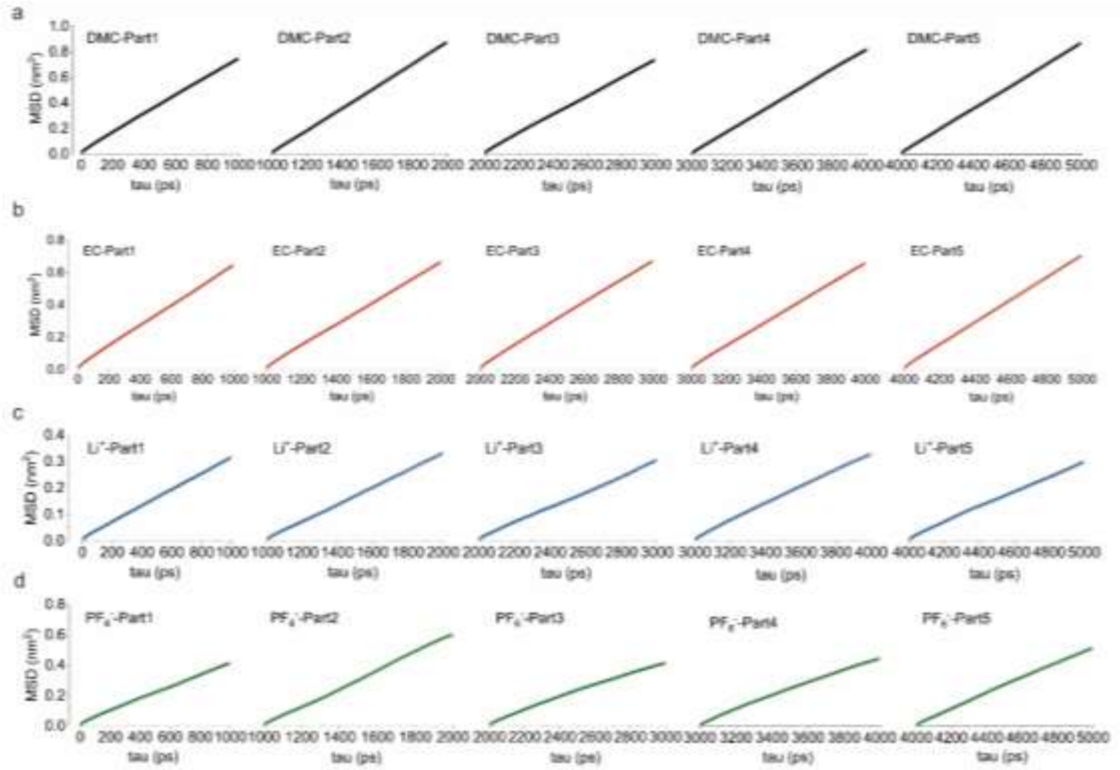

Fig S11. Mean squared displacement (MSD) of all the components in EC-DMC electrolytes at 25 ℃.

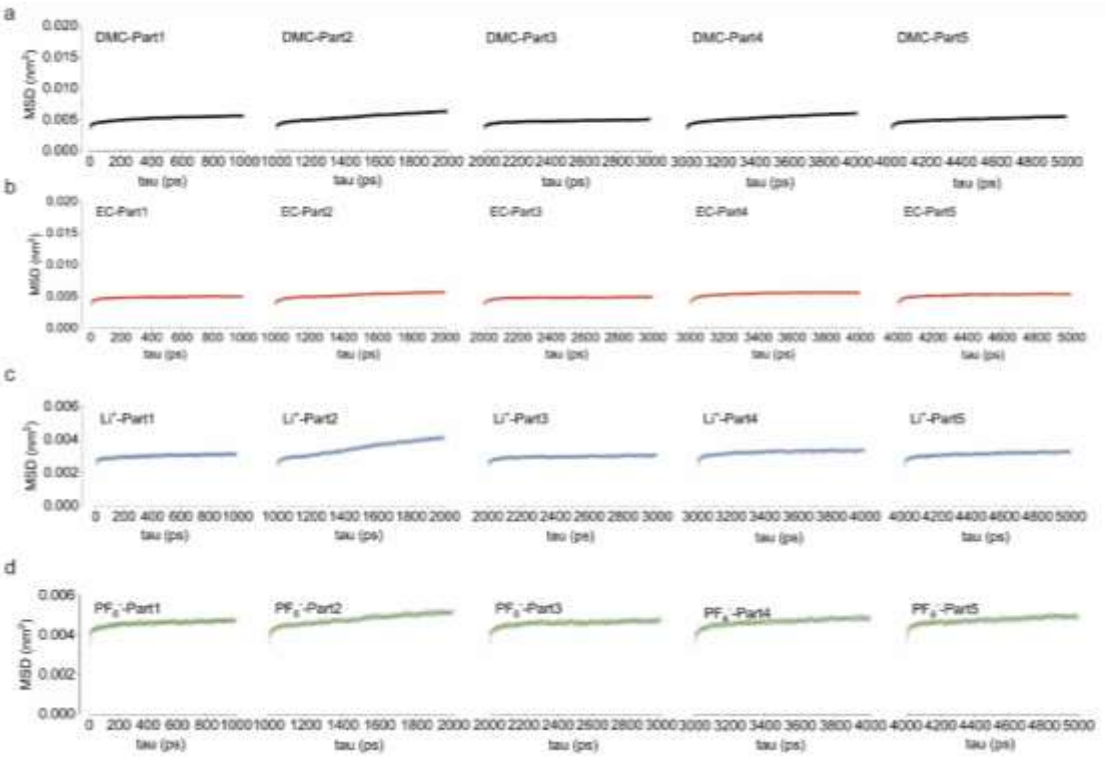

Fig S12. Mean squared displacement (MSD) of all the components in EC-DMC electrolytes at -60 ℃.

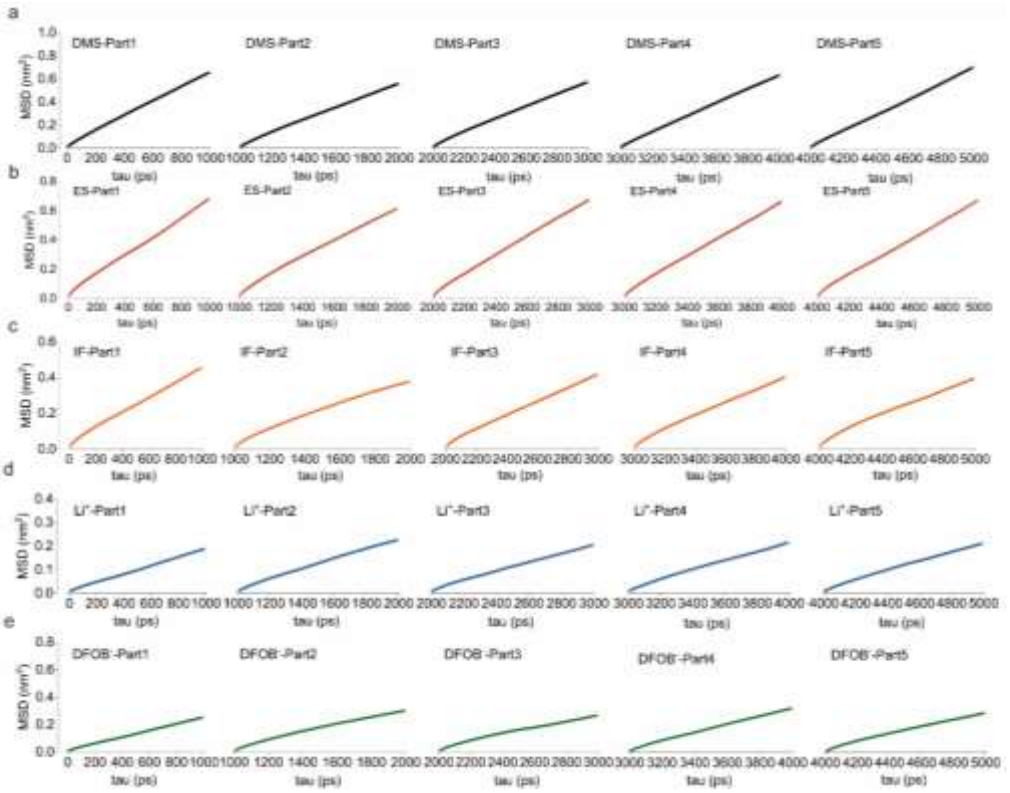

Fig S13. Mean squared displacement (MSD) of all the components in ES-DMS electrolytes at 25 ℃.

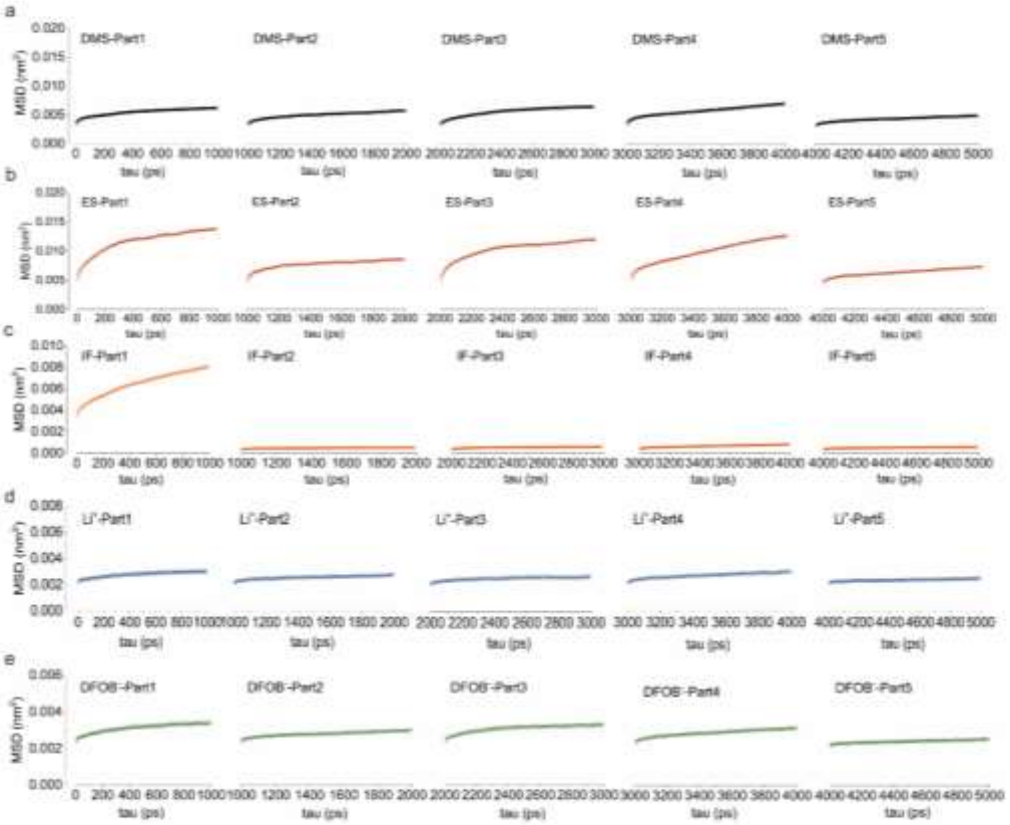

Fig S14. Mean squared displacement (MSD) of all the components in ES-DMS electrolytes at -60 ℃.

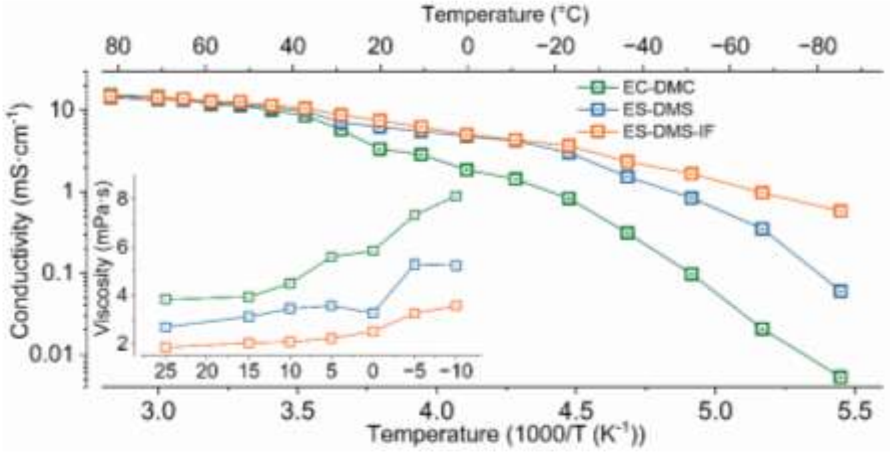

Fig S15. Temperature-dependent ionic conductivity.

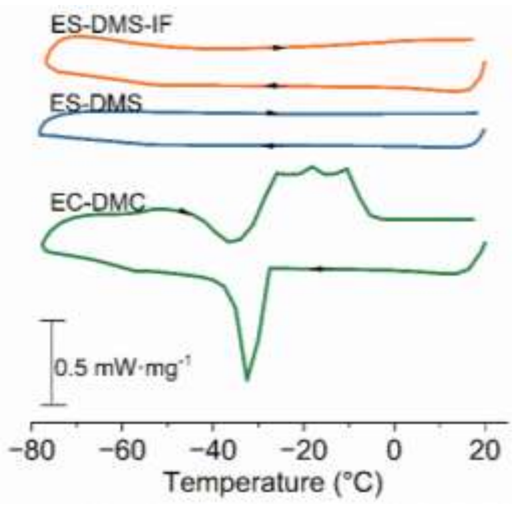

Fig S16. Differential scanner calorimetry (DSC) of three electrolytes.

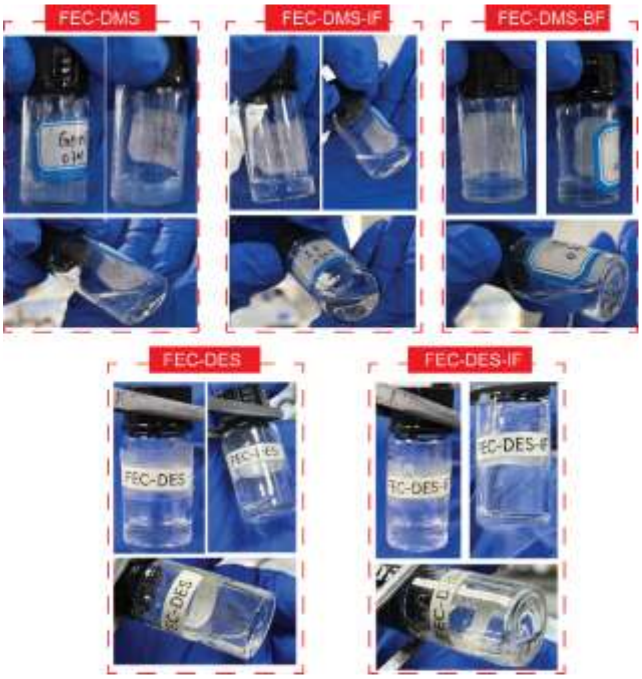

Fig S17. Optical photo of different electrolytes after storage at -110 °C

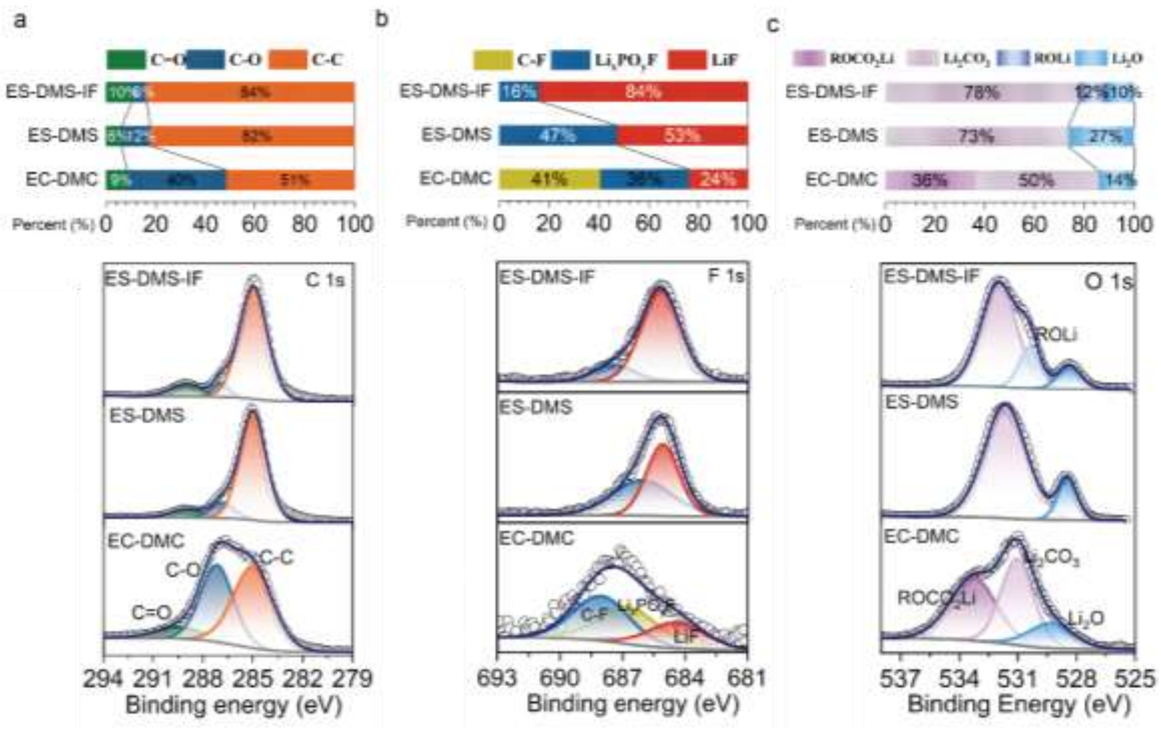

Fig S18. XPS spectra of the Li metal anode after 3 formation cycles in LCO/Li cells. (a) C1*s*; (b) F1*s*; (c) O1*s*.

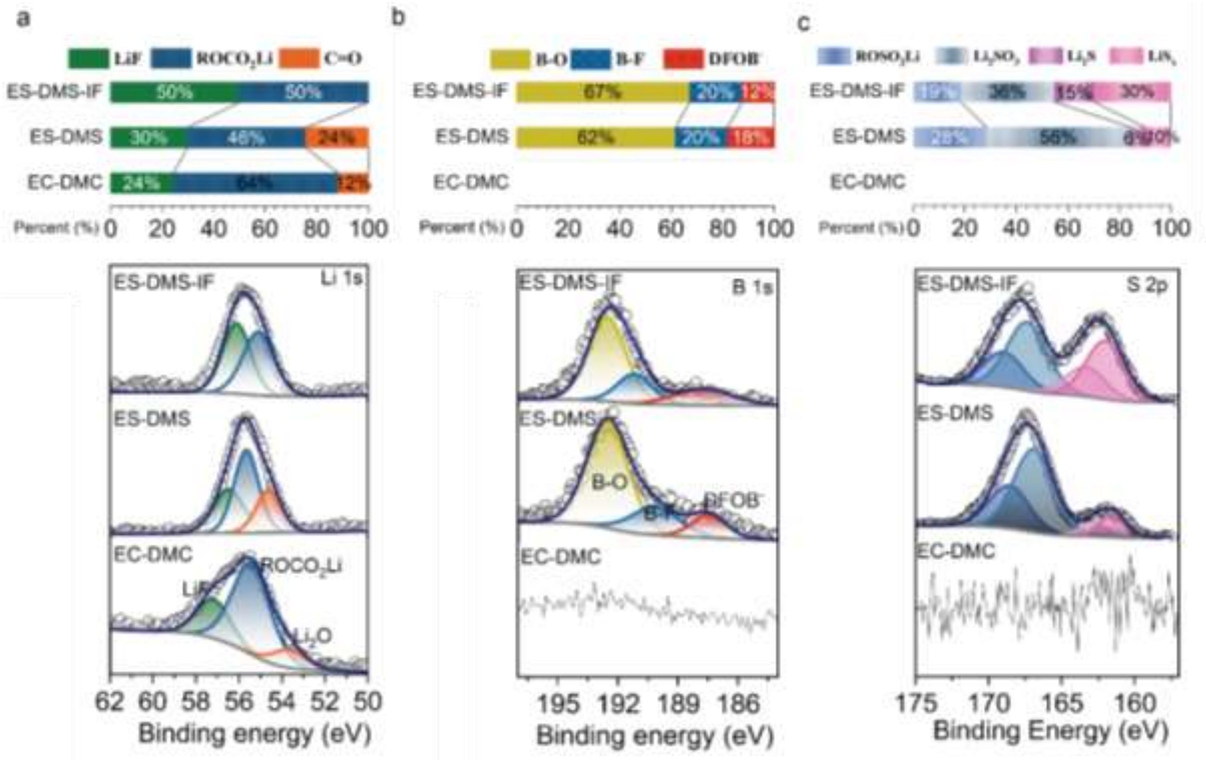

Fig S19. XPS spectra of the Li metal anode after 3 formation cycles in LCO/Li cells. (a) Li1*s*; (b) B1*s*; (c) S2*p*.

Table S3. Summary of fitting information for each peak position of XPS spectrum

Element	Assignment	EC-DMC			ES-DMS			ES-DMS-IF		
		BE (eV)	FWHM (eV)	At (%)	BE (eV)	FWHM (eV)	At (%)	BE (eV)	FWHM (eV)	At (%)
O1s	Li_2O[36-38]	529.31	2.05	14.29	528.45	1.22	26.55	528.35	1.37	10.42
	ROLi[36-38]	–	–	–	–	–	–	530.42	1.18	11.81
	Li_2CO_3[39,40]	531.03	2.93	49.52	531.64	2.65	73.45	531.93	2.31	77.78
	$ROCO_2Li$[41,42]	533.29	2.78	36.19	–	–	–	–	–	–
Li1s	Li_2O[42,43]	53.65	1.94	11.59	54.62	1.44	24.25	–	–	–
	$ROCO_2Li$[42,43]	55.42	2.15	64.24	55.66	1.40	45.98	55.11	1.78	50.06
	LiF[42,43]	57.17	1.90	24.17	56.48	1.72	29.77	56.12	1.60	49.94
F1s	LiF[37,38,40,44]	684.41	3.31	23.68	684.83	2.28	83.97	684.64	1.83	52.53
	Li_xPO_yF[37-39,44]	686.73	3.50	35.67	687.42	2.39	16.03	686.06	3.37	47.47
	C-F[37,38,40,45]	688.01	3.31	40.64	–	–	–	–	–	–
C1s	C-C[37,38,46,47]	285.00	2.92	51.33	285.00	1.91	82.14	285.00	1.97	83.72
	C-O[36-38,44]	271.15	2.41	40.16	286.82	1.72	11.64	286.95	1.11	5.92
	C=O[36-39,43-45]	289.39	3.50	8.51	288.98	2.26	6.22	289.02	2.21	10.35
B1s	Decomposition of $DFOB^{-1}$[26,28,48]	–	–	–	187.43	2.14	12.44	187.70	3.50	18.20
	B-F[26,28,48]				190.13	2.90	20.38	191.16	2.15	20.15
	B-O[26,28,48]	–	–	–	192.52	2.56	67.18	192.54	2.32	61.65
S2p	LiS_x[43]				161.46	2.31	10.23	162.00	3.29	29.93
	Li_2S[43]				162.76	2.31	5.48	163.36	3.29	15.30
	Li_2SO_3[43]				166.90	3.36	55.46	167.37	3.39	36.25
	$ROSO_2Li$[43]				168.58	3.36	28.33	168.99	3.39	18.52

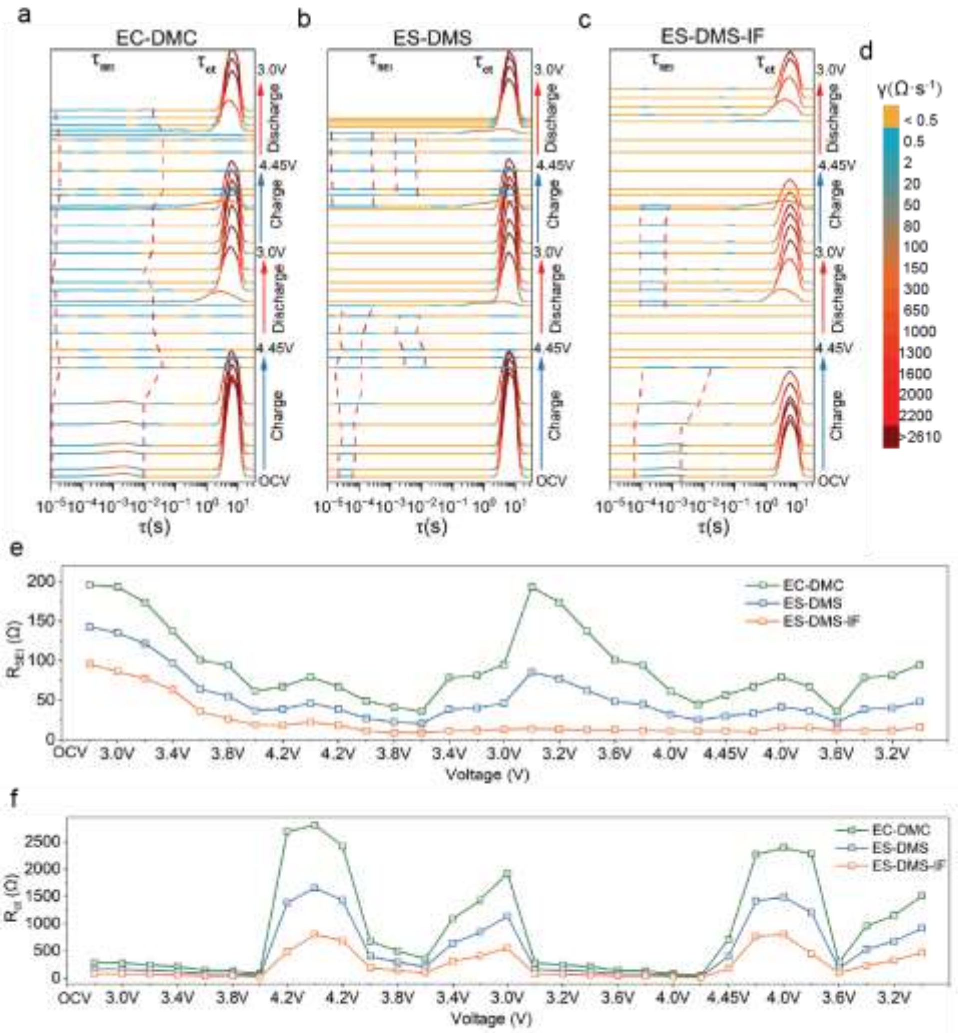

Fig S20. In situ impedance relaxation time distribution (DRT) curves of 4.45 V LCO/Li battery at -10 ℃. (a) EC-DMC, (b) ES-DMS, (c) ES-DMS-IF; (d) impedance scale; (e-f) DRT fitting results for R_{SEI} (e) and R_{ct} (f).

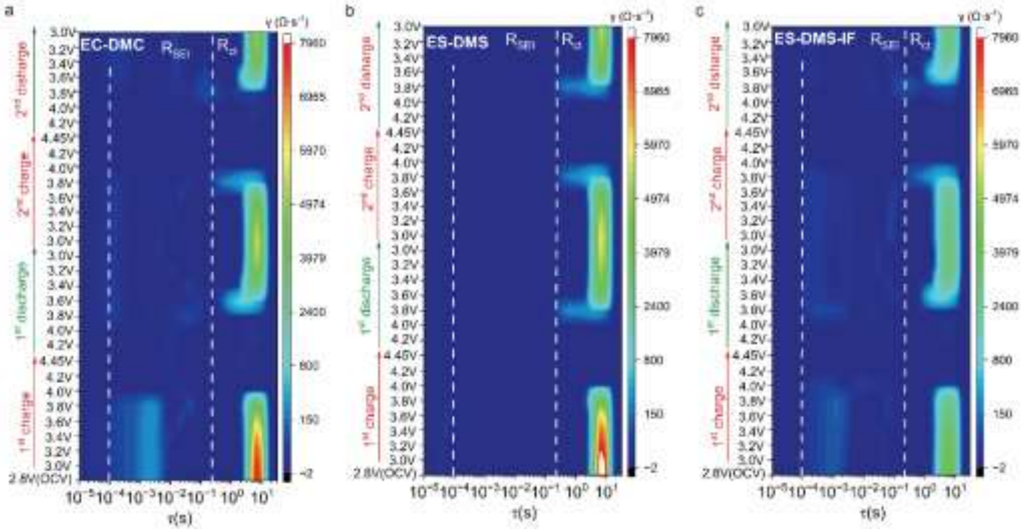

Fig S21. In situ impedance relaxation time distribution (DRT) mapping result. (a) EC-DMC; (b) ES-DMS; (c) ES-DMS-IF

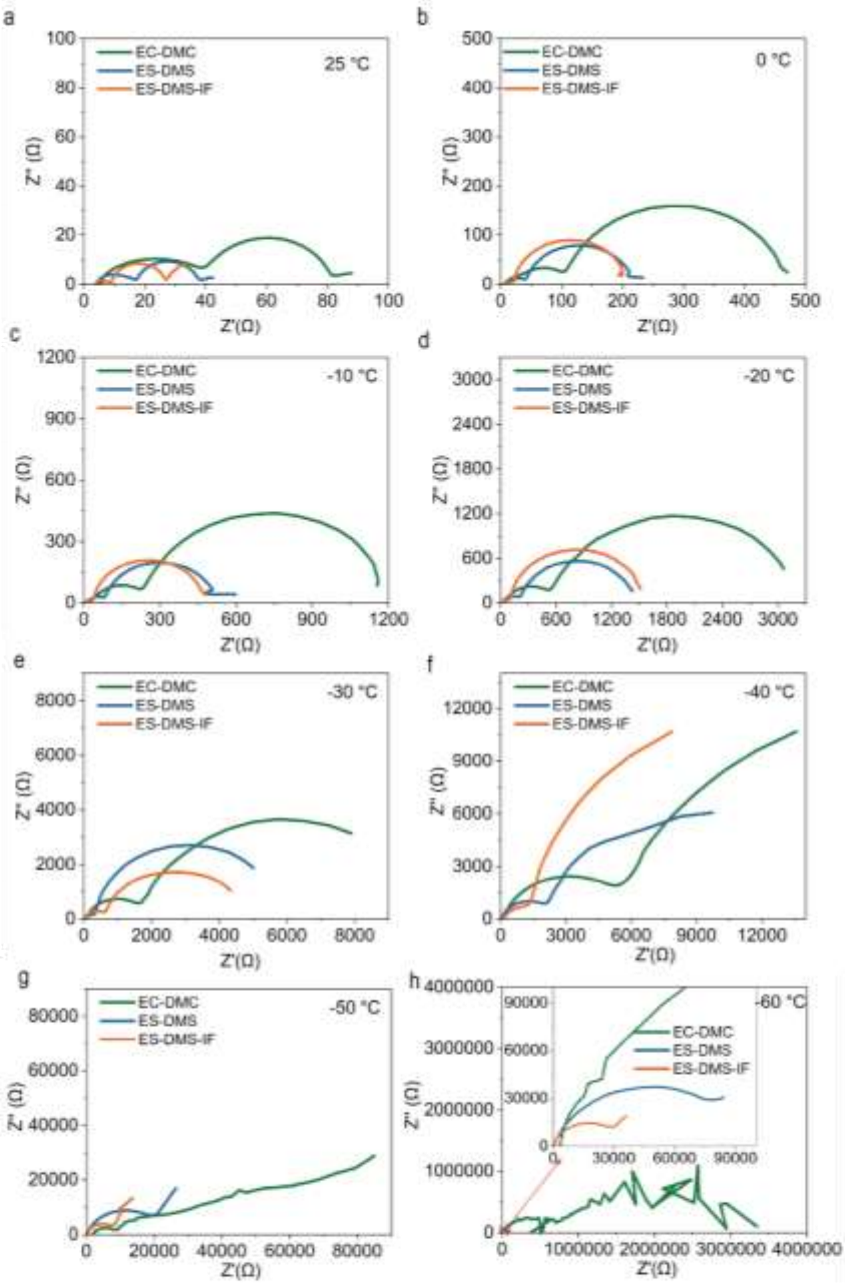

Fig S22. Nyquist curves of the temperature-dependent impedance of LCO/Li batteries. (a) 25 ℃; (b) 0 °C; (c) −10 °C; (d) −20 °C; (e) −30 °C; (f) −40 °C; (g) −50 °C; (h) −60 °C.

As the temperature decreases, the impedance value continues to increase, and the curvature of the second semicircle of the impedance spectrum gradually increases to become a straight line (Fig S15). The Nyquist plot of the impedance spectrum at room temperature consists of two semicircles and a straight line. The first semicircle in the mid-frequency region corresponds to the interface impedance R_{SEI}, and the semicircle in the low-frequency region belongs to the charge transfer impedance R_{ct}. The extremely low-frequency oblique straight line represents the Warburg impedance of Li^+ in solid phase diffusion. The first point where the EIS curve intersects the real part is the ohmic impedance R0 generated by the contact between the electrolyte and the electrode and the diaphragm in the battery. When the temperature drops to 0 ℃, the straight line segment disappears. At -20 ℃, the second semicircle begins to be incomplete and then becomes a straight line at -40 ℃.

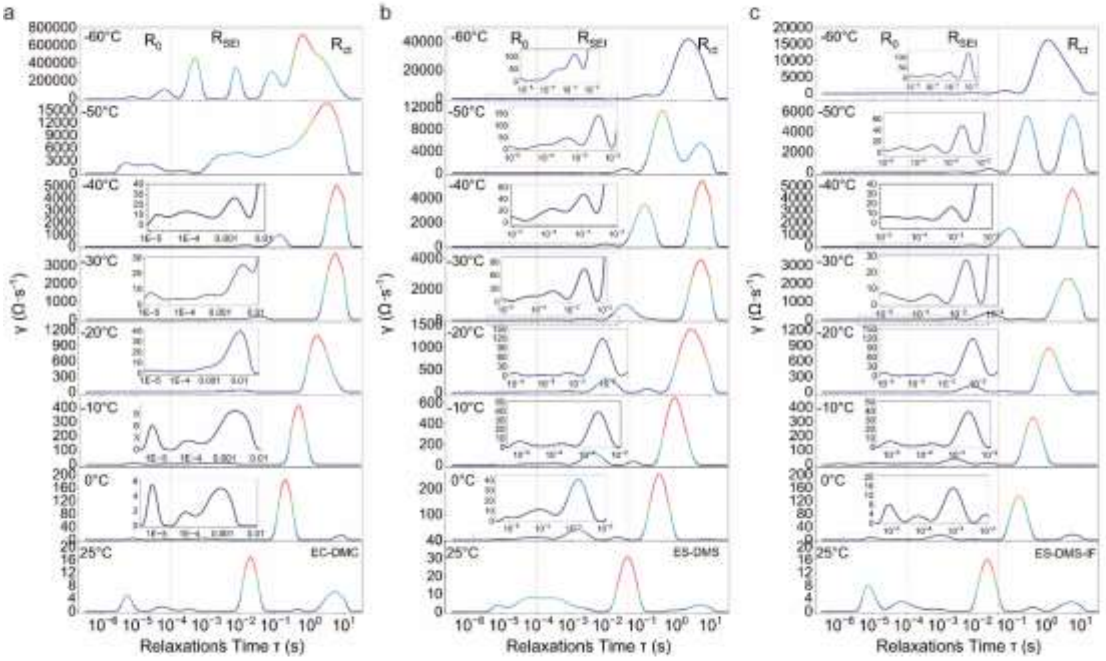

Fig S23. Temperature dependent relaxation time distribution (DRT) curves of 4.45 V LCO/Li battery from -60 ℃ to 25 ℃.

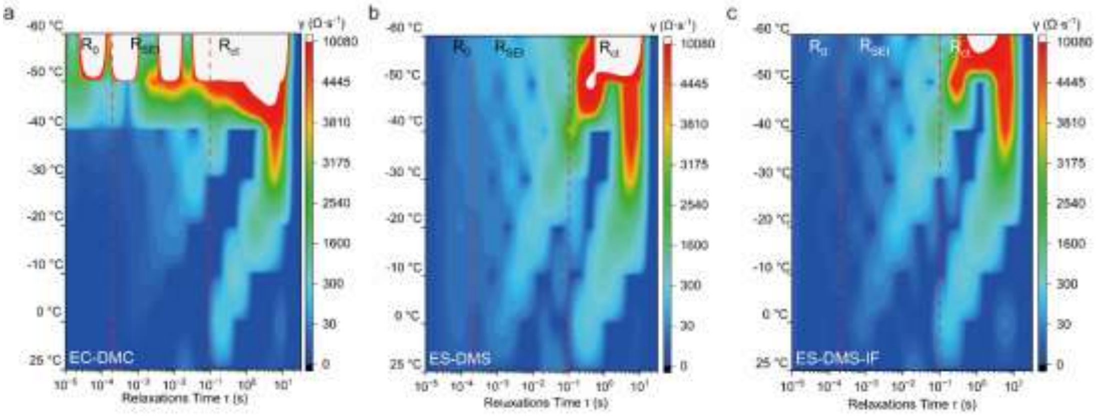

Fig S24. Temperature dependent relaxation time distribution (DRT) mapping result from -60 ℃ to 25 ℃. (a) EC-DMC; (b) ES-DMS; (c) ES-DMS-IF.

Table S4. Impedance fitting data of LCO/Li battery at different temperatures

Temp	EC-DMC			ES-DMS			ES-DMS-IF		
	R_0	R_{SEI}	R_{ct}	R_0	R_{SEI}	R_{ct}	R_0	R_{SEI}	R_{ct}
25 ℃	3.593	36.52	46.18	3.996	5.347	18.32	4.288	12.4	21.85
0 ℃	4.821	123.6	380.1	2.917	38.55	189.3	5.771	81	182.5
-10 ℃	6.882	368.3	1020	4.815	169.9	446.5	6.733	265	462.9
-20 ℃	12.9	1014	2899	7.881	619.4	1521	9.196	608.8	1311
-30 ℃	27.76	2971	9520	12.85	1266	6094	14.9	1221	4328
-40 ℃	75.54	9473	60394	20.31	4811	34798	24.3	3534	17273
-50 ℃	99	18733	233000	31.36	7333	53280	41.77	9008	40500
-60 ℃	NA	NA	NA	52.7	42089	642000	53.53	32080	401800

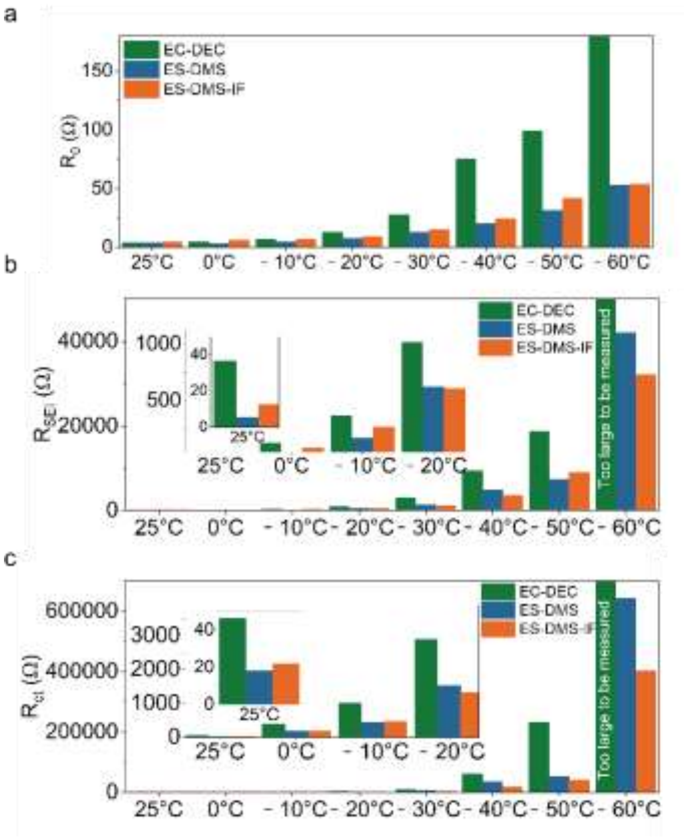

Fig S25. Temperature dependent resistance fitting result. (a) Contact impedance R_0; (b) Interface impedance R_{SEI}; (c) Charge transfer impedance R_{ct}.

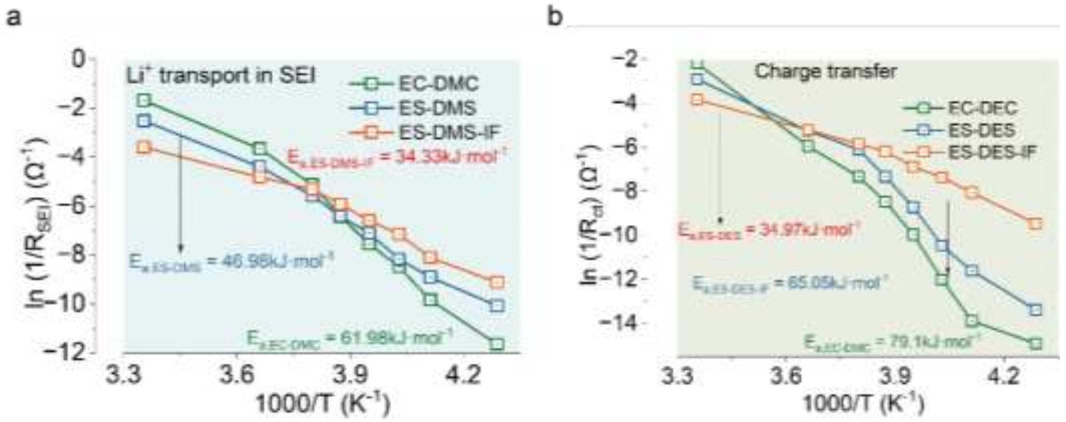

Fig S26. Analysis of temperature-dependent dynamic behavior, Arrhenius curve of impedance variation with temperature form 25°C to -40°C. (a) Interface impedance R_{SEI}; (b) Charge transfer impedance R_{ct}.

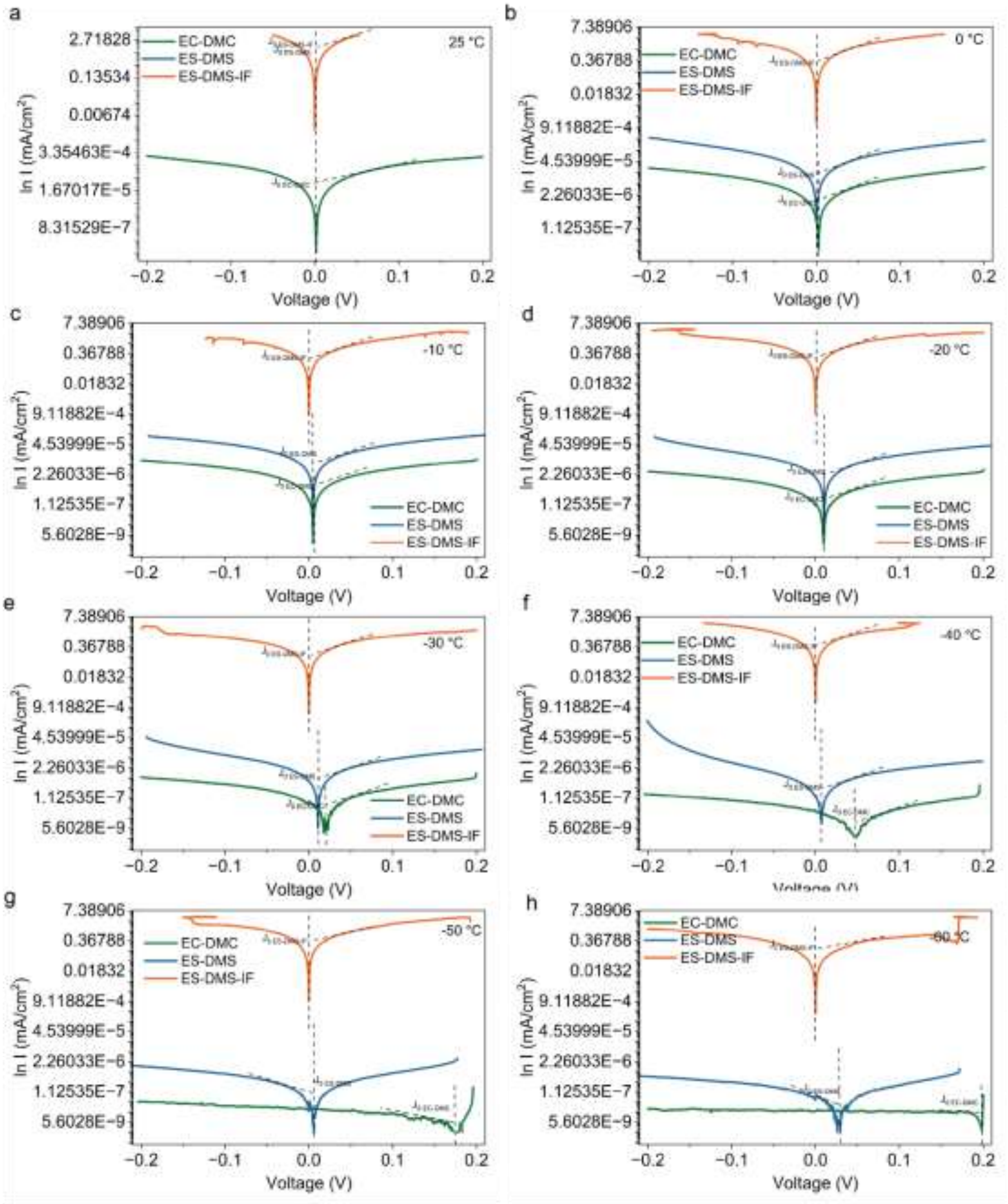

Fig S27. Temperature-dependent Tafel curve of Li/Li symmetric batteries. (a) 25 ℃; (b) 0 ℃; (c) −10 °C; (d) −20 °C; (e) −30 °C; (f) −40 °C; (g) −50 °C; (h) −60 °C.

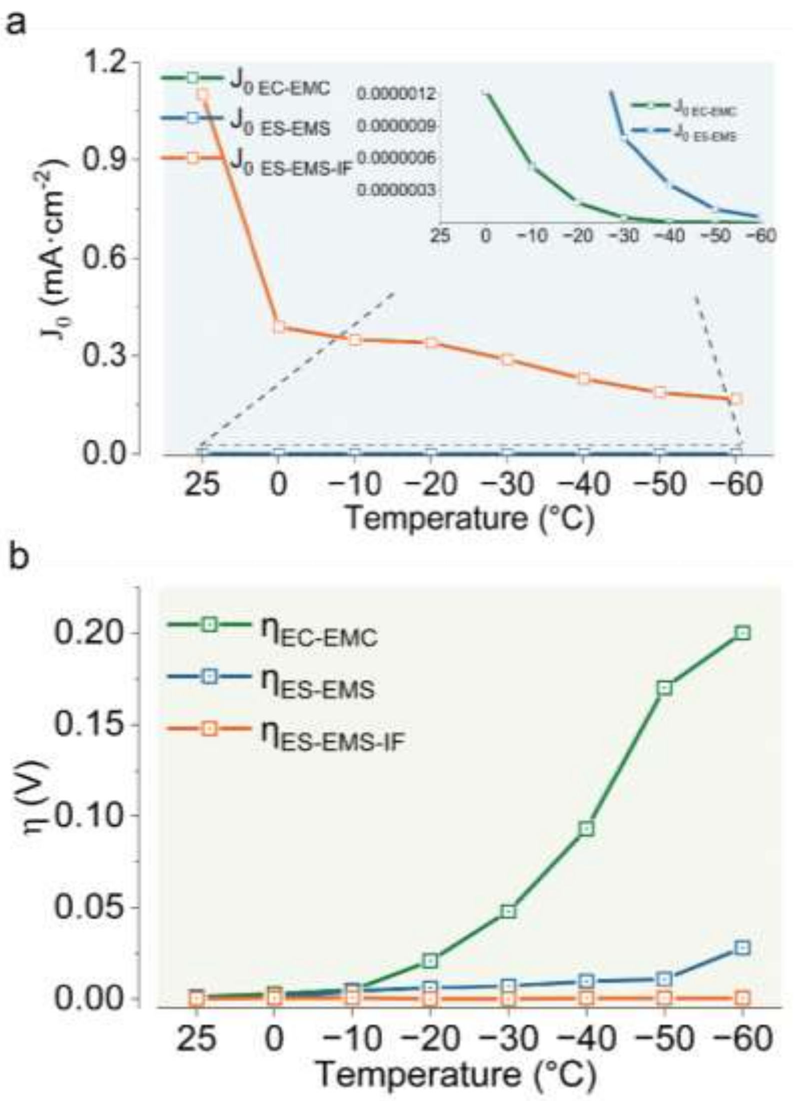

Fig S28. The fitting results of the temperature-dependent Tafel curve parameters of Li/Li symmetric batteries. (a) The trend of exchange current density changing with temperature; (b) The trend of overpotential changing with temperature.

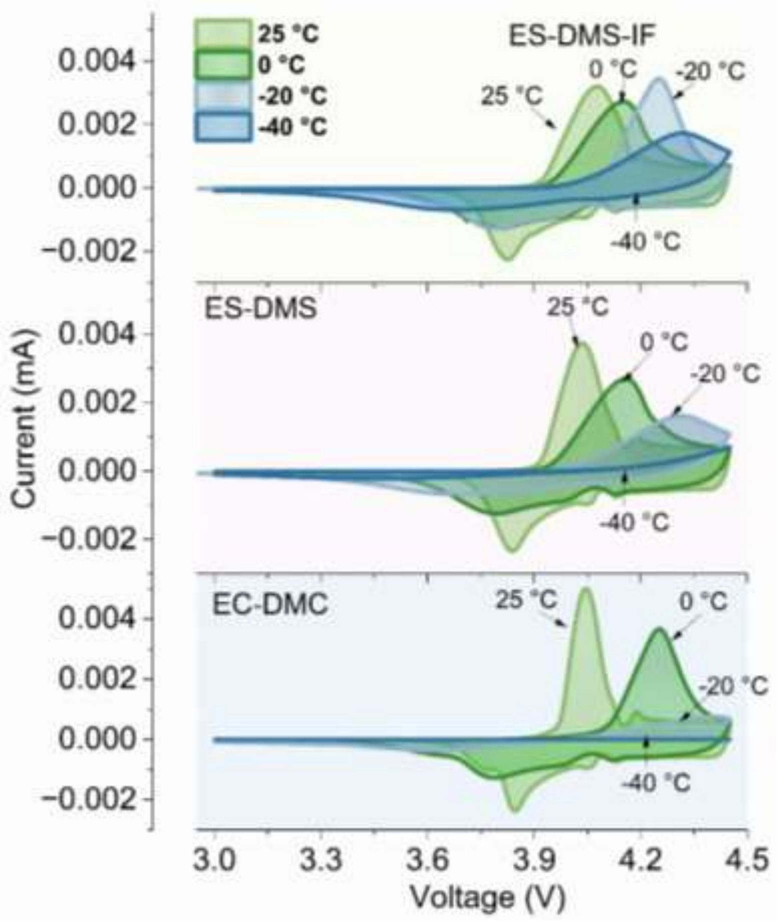

Fig S29. Temperature-dependent CV curves of LCO/Li batteries.

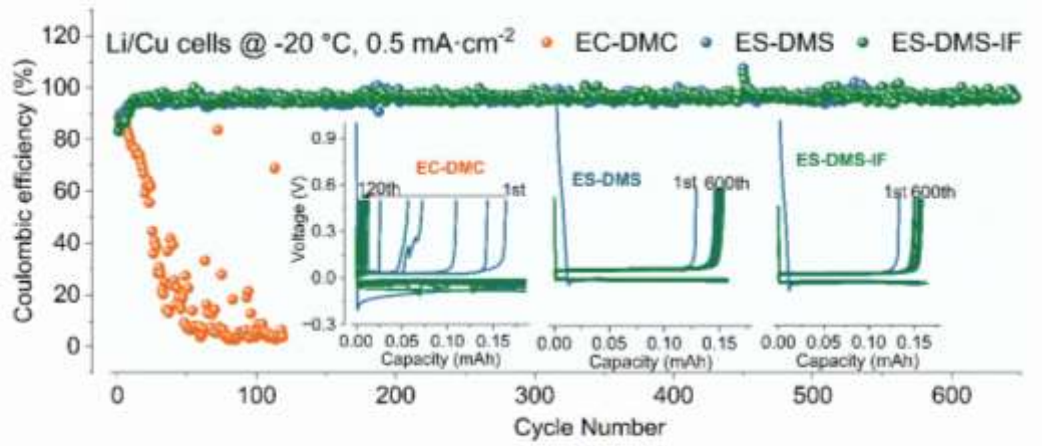

Fig S30. Coulombic efficiency of Li/Cu batteries with charge and discharge profile inserted.

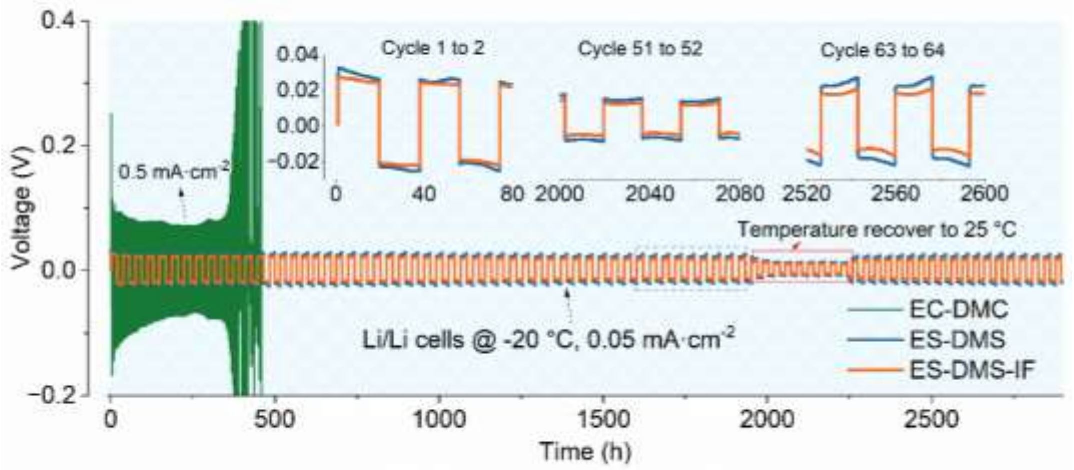

Fig S31. Cycling time-voltage profiles of Li/Li symmetric batteries.

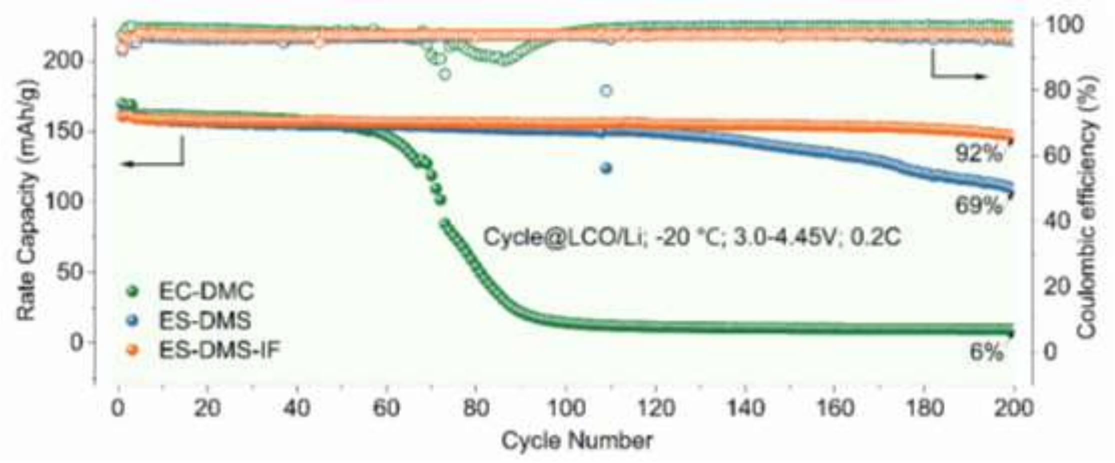

Fig S32. Cycle performance of LCO/Li battery at -20℃.

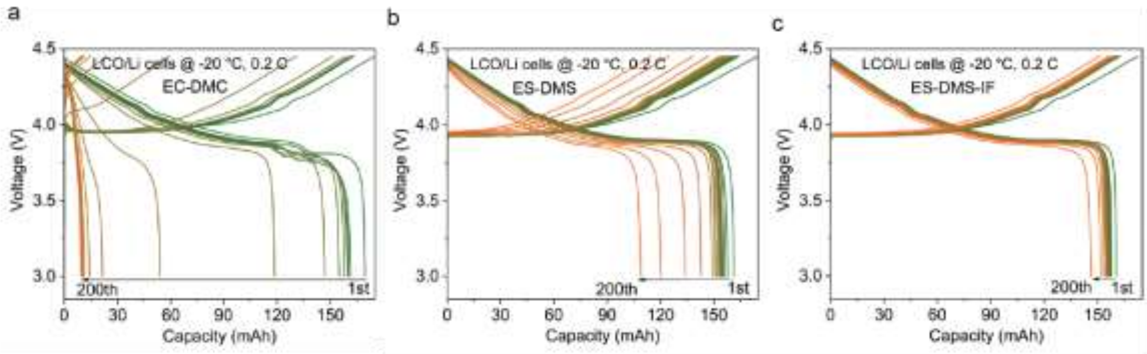

Fig S33. Capacity-voltage profile of LCO/Li batteries at -20℃. (a) EC-DMC; (b) ES-DMS; (c) ES-DMS-IF.

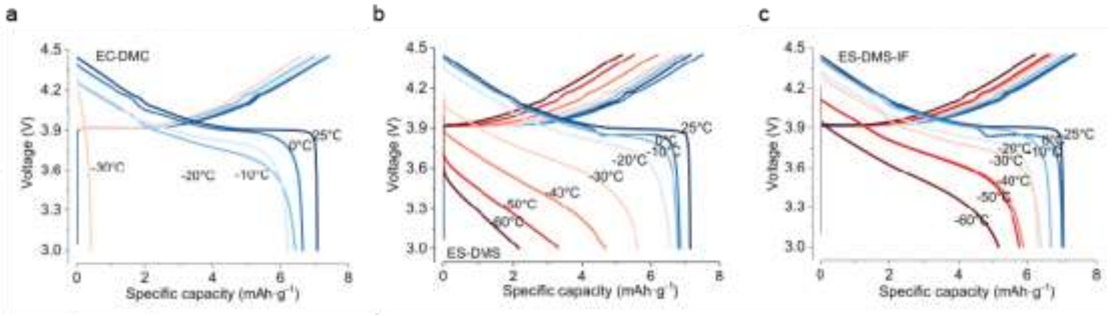

Fig S34. Temperature dependent capacity-voltage profiles of 7.5Ah LCO/Li pouch cell within -60 ℃ to 25 ℃. (a) EC-DMC; (b) ES-DMS; (c) ES-DMS-IF.

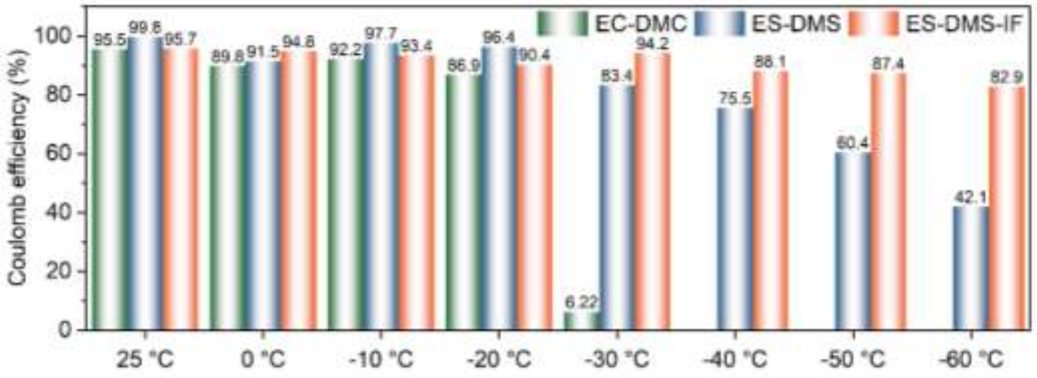

Fig S35. Coulombic efficiency of LCO/Li battery during temperature dependent cycles.

Table S5. Capacity and Coulombic efficiency of LCO/Li pouch cells during temperature dependent charge-discharge cycles.

Temp	EC-DMC			ES-DMS			ES-DMS-IF		
	Charge Capacity (Ah)	Discharge Capacity (mAh)	CE %	Charge Capacity (Ah)	Discharge Capacity (mAh)	CE %	Charge Capacity (Ah)	Discharge Capacity (mAh)	CE %
25 ℃	167.9	160.3	95.5	162.5	162.1	99.8	166.8	159.7	95.7
0 ℃	168	150.8	89.8	170.1	155.6	91.5	168	159.3	94.8
-10 ℃	158.2	145.9	92.2	157.9	154.3	97.7	162.5	151.8	93.4
-20 ℃	162.5	141.2	86.9	154.5	148.9	96.4	158.6	143.3	90.4
-30 ℃	149.4	9.3	6.2	153.4	127.9	83.4	154.2	145.3	94.2
-40 ℃				140.6	106.2	75.5	151.5	133.5	88.1
-50 ℃				125	75.5	60.4	149.9	131	87.4
-60 ℃				117	49.2	42.1	141.2	117	82.9

Table S6. 7.5Ah Pouch cell Information

Cathode		Anode		Electrolyte dosage	Work Voltage	Thickness	Weight
Materials	Area Density	Materials	Thickness				
LCO	280g m^{-2}	Li metal	30μm	11.25g	3.95 V	7.2 cm	65.6 g

Table S7. Conductivity of different electrolyte

Temperature (℃)	1000/T (K^{-1})	Conductivity (mS cm^{-1})		
		EC-DMC	ES-DMS	ES-DMS-IF
80	2.82885	15.32	14.32	14.88
60	2.9985	14.78	13.53	14.32
50	3.09119	13.73	13.32	13.98
40	3.18979	11.81	12.34	12.89
30	3.29489	11.51	11.78	12.76
20	3.40716	10.03	10.89	11.53
10	3.52734	8.613	9.78	10.67
0	3.65631	5.789	7.14	8.76
-10	3.79507	3.365	6.285	7.54
-20	3.94477	2.867	5.549	6.27
-30	4.10678	1.865	4.842	5.03
-40	4.28266	1.432	4.255	4.35
-50	4.47427	0.827	3.02	3.68
-60	4.68384	0.312	1.53	2.35
-70	4.914	0.09762	0.84	1.67
-80	5.16796	0.02067	0.35	0.97
-90	5.44959	0.00527	0.06	0.58

Table S8. Viscosity of different electrolyte

Temperature (℃)	Viscosity (mPa·s)		
	EC-DMC	ES-DMS	ES-DMS-IF
-10	8.13	5.25	3.59
-5	7.34	5.3	3.27
0	5.86	3.28	2.53
5	5.61	3.59	2.24
10	4.51	3.47	2.09
15	3.95	3.13	2.05
25	3.86	2.71	1.87

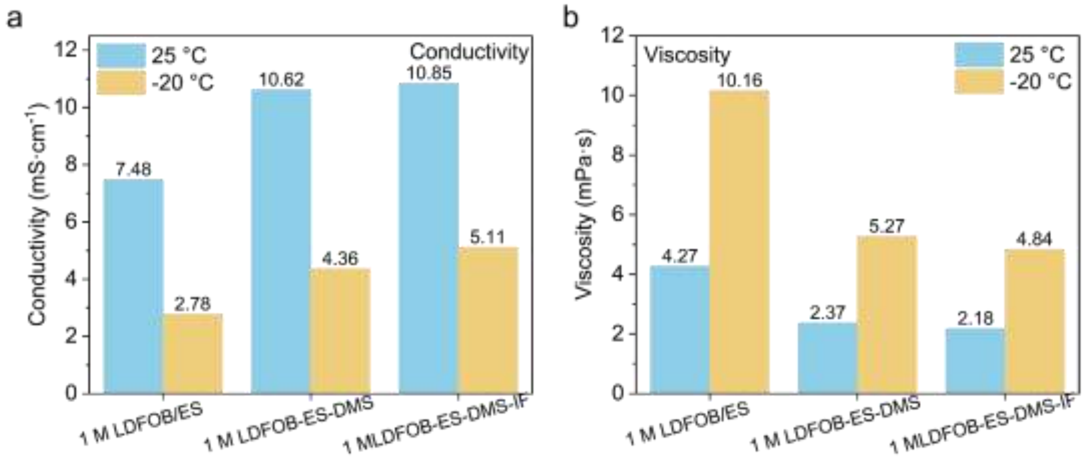

Fig S36. Ionic conductivity and viscosity of the three sulfite-based electrolytes under -20 ℃ and 25 ℃. (a) Conductivity; (b) Viscosity.

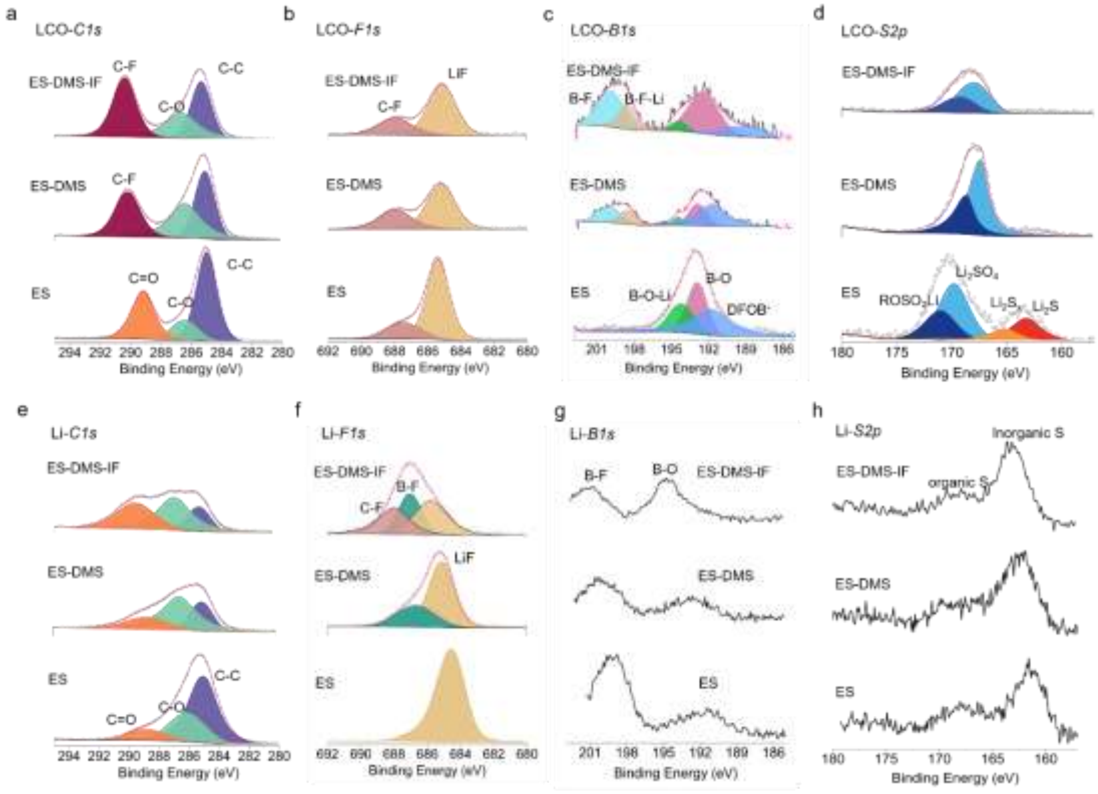

Fig S37. XPS analysis for the interphase chemical information of cathode and anode with the suggested three sulfite-based electrolytes. The CEIs composition on the surface of LCO cathode (a-d) and the SEIs species on the surface of Li anode (e-h) after formation cycles. (a,e) C*1s*; (b,f) F*1s*; (c,g) B*1s*; (d,h) S*2p*.

Table S9. Summary of fitting information for each peak position of XPS spectrum for the suggested three sulfite-based electrolytes.

Group	Peak	Assignments	LDFOB-ES			LDFOB-ES-DMS			LDFOB-ES-DMS-IF		
			BE (eV)	FWHM (eV)	At(%)	BE (eV)	FWHM (eV)	At (%)	BE (eV)	FWHM (eV)	At(%)
Cathode	C1*s*	C-C	285.00	1.6	50.16	285.00	1.57	35.42	285.00	1.64	32.39
		C-O-C	286.47	2.07	13.90	286.31	2.40	34.25	286.37	2.60	28.66
		C=O	289.14	1.81	30.69						
		C-F				290.09	1.94	30.32	290.17	1.85	38.95
	F1*s*	LiF	685.39	1.61	76.08	685.20	2.23	66.73	685.11	2.00	70.71
		C-F	687.48	2.49	23.92	687.90	2.23	33.27	687.93	2.42	29.29
	S2*p*	Li_2S	163.22	3.35	17.65						
		Li_2S_x	165.18	3.35	18.70						
		Li_2SO_4	169.78	3.53	31.81	167.23	2.23	49.97	167.81	3.55	50.03
		$ROSO_2Li$	171.00	3.47	31.83	168.53	2.23	50.03	169.11	3.55	49.97
	B1*s*	$DOBF^-$	191.76	2.90	40.02	191.56	2.63	42.33	189.66	3.55	8.65
		B-O	192.90	1.91	36.84	192.75	1.73	16.43	192.21	2.99	32.11
		B-O-Li	194.20	2.42	23.15	194.34	1.73	5.98	194.19	1.59	12.78
		B-F-Li				198.44	1.58	15.20	198.49	1.49	17.88
		B-F				199.92	2.32	20.06	199.82	2.55	28.58
	C1*s*	C-C	285.00	2.30	58.71	285.00	1.78	26.14	285.00	1.83	19.17
		C-O-C	286.16	2.90	29.42	286.51	2.74	55.35	286.67	2.73	34.68
		C=O	288.81	2.98	11.88	288.63	3.21	18.51	289.38	2.80	46.16
	F1*s*	LiF	685		100%	685.06		67.65	685.76	2.39	39.70
		B-F				686.68		32.35	687.02	1.60	29.95
		C-F							687.98	2.12	30.35
	S2*p*	Li_2S	160.95			162.36		162.56			
		Li_2S_x	162.05			166.90		163.66			
		Li_2SO_4	165.33			169.39		167.28			
		$ROSO_2Li$	168.23			170.49		169.01			

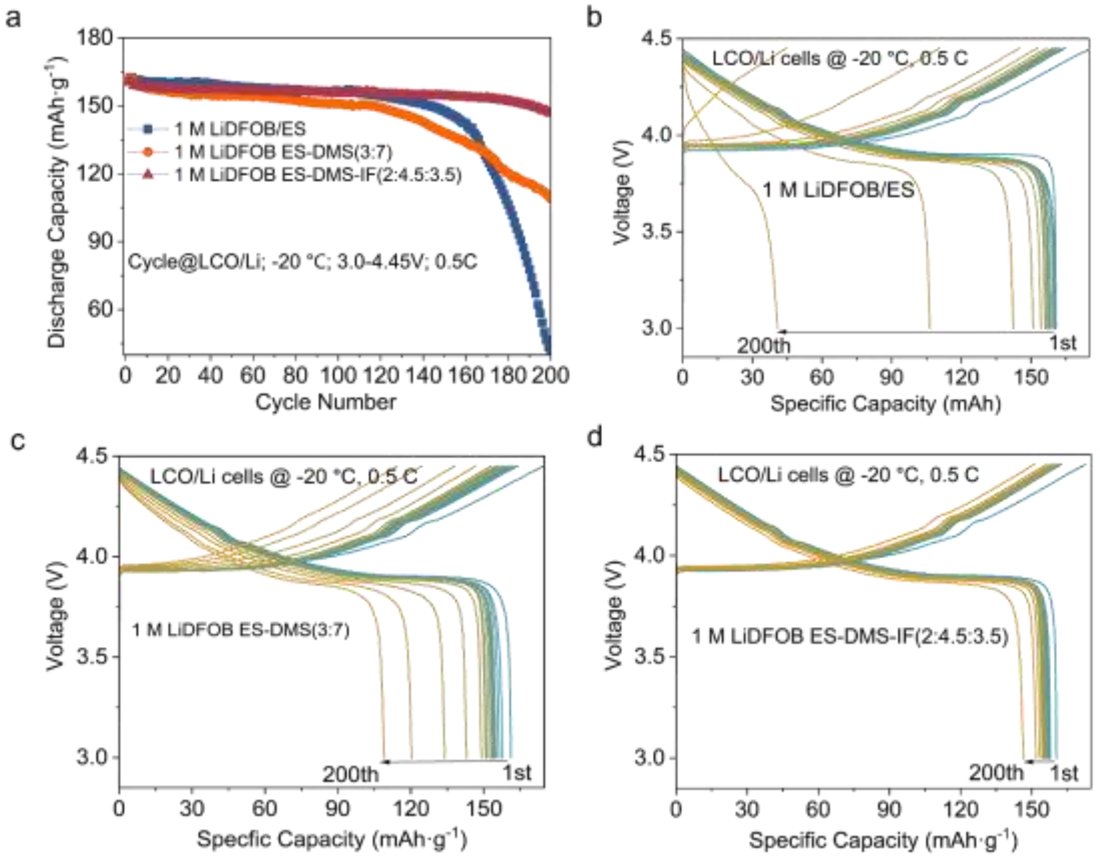

Fig S38. Cycling performance of LCO/Li coin cell under -20 °C with different electrolyte. (a) Cycling curve; (b-d) Charge-Discharge curve for 1M LiDFOB in ES (b), 1M LiDFOB in ES-DMS (c) and 1M LiDFOB ES-DMS-IF (d)

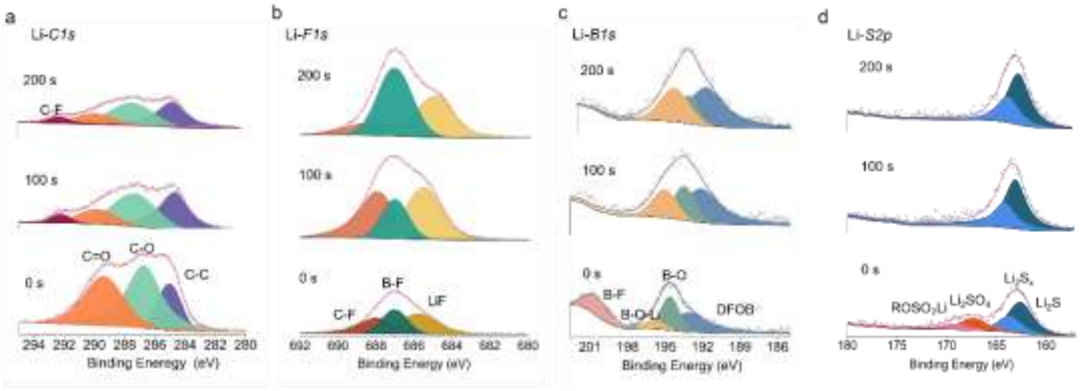

Fig S39. In-depth XPS analysis for the interphase chemical information Li metal anode with the ES-DMS-IF electrolyte. (a) C *1s*; (b) F *1s*; (c) B *1s*; (d) S *2p*.

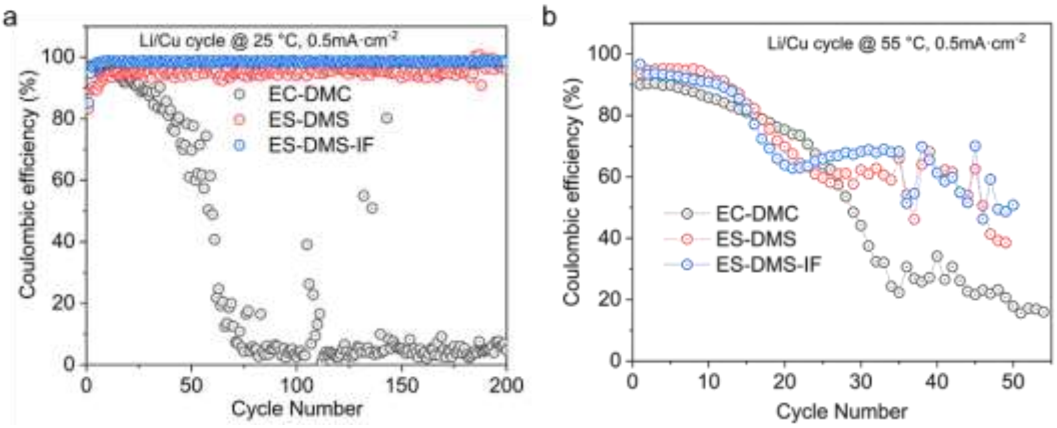

Fig S40. Coulombic efficiency of Li/Cu cells during cycling under 25 °C (a) and 55 °C (b).

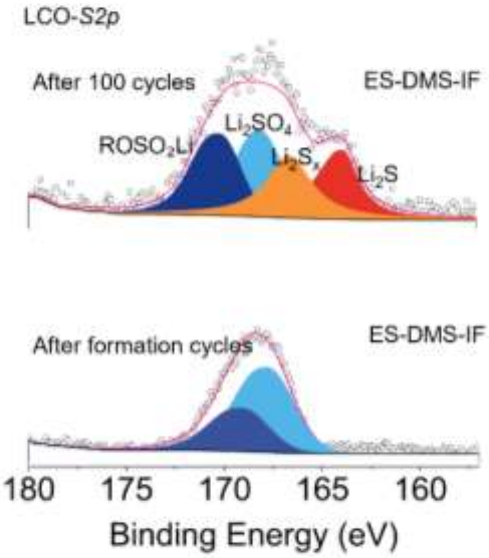

Fig S41. XPS analysis of S2*p* for LCO cathode with the ES-DMS-IF electrolyte before and after long cycles.

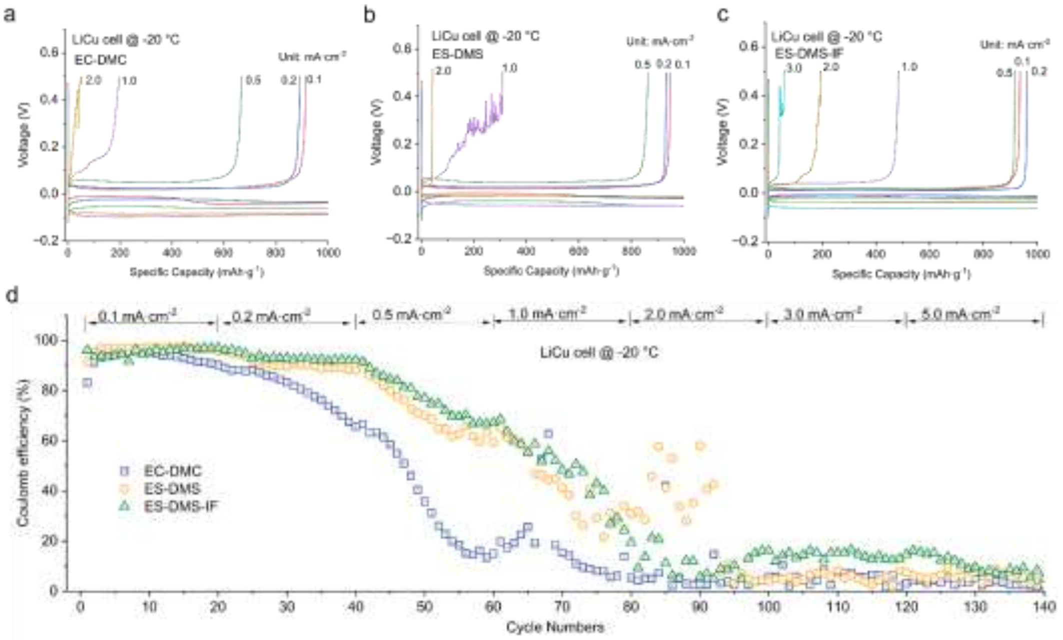

Fig S42. Low temperature cycling performance under different current density. (a) Coulomb efficiency of the three electrolytes; (b-d) Charge and discharge curve for EC-DMC electrolyte (b), ES-DMS electrolyte (c), and ES-DMS-IF electrolyte (d).

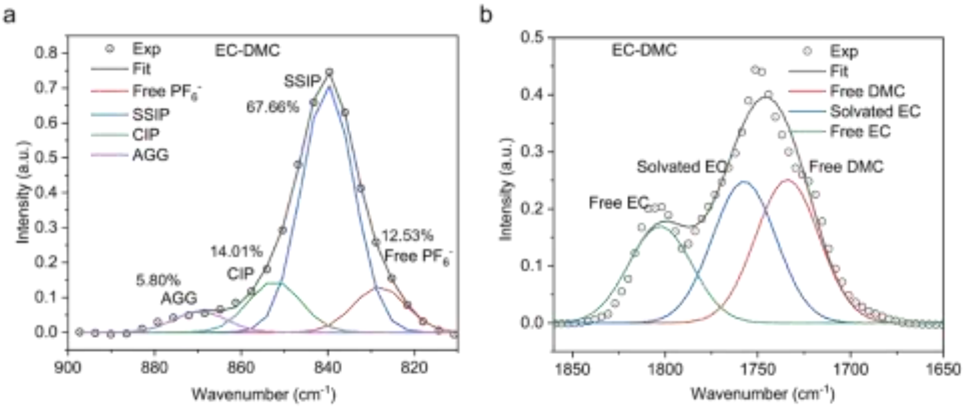

Fig S43. FTIR spectrum of EC-DMC electrolyte. (a) P-F band; (b) C=O band.

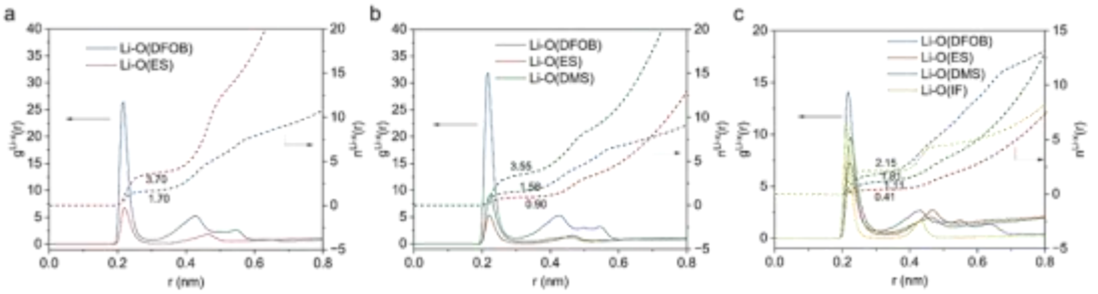

Fig S44. The calculated radial distribution functions (g(r)) and coordination numbers (n(r)) for LiDFOB in ES electrolyte (a), ES-DMS electrolyte (b), and ES-DMS-IF electrolyte (c).

METHODS

Electrolyte preparation and battery fabrication

The EC-DMC electrolyte was prepared by dissolving 1M $LiPF_6$ in a mixture of common carbonate solvents (EC: DMC = 3:7 by vol%). Test electrolytes were prepared by dissolving 1M LiDFOB in the mixture of sulfite solvents and carboxylate solvents, with ES: DMS = 3:7 (by vol%) and ES: DMS: IF=2:4.5:3.5 (by vol%) for ES-DMS electrolyte and ES-DMS-IF electrolyte, respectively. All the electrolytes were prepared in the glovebox with H_2O and O_2 content <0.01 ppm (Shanghai Mikrouna Electromechanical Technology Co., Ltd, Universal).

The standard LCO/Li pouch cell with an electrochemical window of 3-4.45V was fabricated by injecting 11.25g electrolyte into a 7.5Ah dry pouch cell with thin Li metal[69] (supplied by Wanxiang A123 Co., Ltd.) within an argon-filled glovebox. The fabrication process involved an initial sealing and formation stage, followed by venting the gas generated during formation from the gasbag compartment, and finalization through secondary sealing to complete the electrochemical cell preparation.

LCO/Li, and Li/Li coin cells have been made with $LiCoO_2$ cathode, and lithium anode respectively (all sourced by Guangdong Canrd Ltd.) in a glovebox filled with argon, with specific surface area of 1.88 m^2 g^{-1} for LCO cathode. (Measured by a nitrogen adsorption-desorption isotherm (JW-BK200C, Beijing JWGB SCI & TECH).

Electrolyte characteristic

The conductivity of the electrolytes was determined with a conductivity meter (Mettler Toledo, Shanghai, China) in a cold trap containing a mixture of ethyl alcohol and liquid nitrogen whose temperature was adjusted by varying the ratio of the two substances. FTIR spectra were acquired using a Fourier Transform Infrared Spectrometer (Nicolet, iS50 FT-IR, Thermo Scientific, KBr tablet, wavenumber 4000-600 cm^{-1}). Raman spectra were acquired with a WITEC alpha 300R Raman system (532 nm laser, laser power 2 mW). The electrolyte was storage at the onset

temperature with the same temperature controlling device of conductivity before FTIR and Raman test.

Electrochemical measurements

The charge/discharge behaviors of the batteries (coin cells and pouch cells) were tested using a battery test system (CT-4008T-5V6A, CT-4008T-5V10mA, Neware, Shenzhen, China, and CT2001A, Wuhan Land Electronics Co. Ltd). Batteries to be tested were placed in the chamber (GMC-71, Espec, Guangzhou, China) and brought to an appropriate onset temperature for the desired test Electrical impedance spectroscopy was performed over a frequency range from 0.01 Hz to 100K Hz on an electrochemical work station (Solartron 1455A, Solartron Group, UK) with an amplitude of 10 mV. Cyclic voltammetry profiles obtained in the potential range from 3.0 V ~ 4.45 V (vs Li/ Li^+) with a scan rate of 0.2 mV/s and Tafel test (performed in lithium symmetrical coin cell within a voltage range of -0.2~0.2V with a scan rate of 0.5mV/s) were also performed on Solartron electrochemical station.

Material characterizations

X-ray photoelectron spectroscopy system equipped with a vacuum transfer vessel accessory (XPS, Ulvac-Phi, PHI Versaprobe 4, KW-ST lab, www.kewei-scitech.com) was used to analyze the interface composition on the surface of the $LiCoO_2$ cathode. The spectra were processed using commercial software (Thermo Avantage), and the atomic concentrations were determined using the relative sensitivity factors (Scofield factors). DSC tests were conducted by the thermal analyzer (Navi Innovation Co. Ltd, navi-sci.cn). The conductivity test was conducted by the conductivity meter (Mettler, SevenCompact S230). The viscosity test was conducted by the rotational rheometer (AMETEK Brookfield, DVNXLVCJG)

Computational methods

The DFT calculations were performed using Gaussian 16 at the B3LYP/6-311G level for geometric optimizations, vibrational frequency analysis, and bond energy

scanning, with dispersion corrections applied via the GD3BJ method. Molecular van der Waals volumes were determined using Multiwfn. Classical molecular dynamics (cMD) simulations employed GROMACS 2024.6, where electrolyte systems (carbonate-based: 5 $LiPF_6$ /22 EC/40 DMC; sulfite-based: 5 LiDFOB/13 ES/25 DMS/15 IF) were packed via PACKMOL with 2.5 Å tolerance at initial density 1.2 g/cm^3. GAFF force fields generated by Sobtop incorporated 0.8 charge scaling for ions. Simulations utilized 1 fs timestep, PME electrostatics, and dispersion-corrected van der Waals interactions under periodic boundary conditions.

After energy minimization (conjugate gradient, 50 kJ·mol^{-1}·nm^{-1} force threshold), systems underwent 5 ns NPT equilibration at 25℃/1 bar using V-rescale/Berendsen coupling, reaching densities of 1.267 g/cm^3 (carbonate) and 1.269 g/cm^3(sulfite). Production runs (50 ns NVT) followed, with -60℃ simulations incorporating 850 ps annealing from 25℃ prior to equilibration, yielding elevated densities of 1.328 g/cm^3 and 1.307 g/cm^3, respectively. Trajectory analysis included radial distribution functions and mean square displacement calculations via VMD.

Reference

1. Maier, J. (1987). Defect Chemistry and Conductivity Effects in Heterogeneous Solid Electrolytes. Journal of The Electrochemical Society *134*, 1524. 10.1149/1.2100703.
2. Shen, K.-H., and Hall, L.M. (2020). Effects of Ion Size and Dielectric Constant on Ion Transport and Transference Number in Polymer Electrolytes. Macromolecules *53*, 10086-10096. 10.1021/acs.macromol.0c02161.
3. Chu, W., Qin, J., and de Pablo, J.J. (2018). Ion Distribution in Microphase-Separated Copolymers with Periodic Dielectric Permittivity. Macromolecules *51*, 1986-1991. 10.1021/acs.macromol.7b02508.
4. Zhang, C., Wang, Z., Zhu, H., Zhang, Q., and Zhu, S. (2024). Dielectric Gels with Microphase Separation for Wide-Range and Self-Damping Pressure Sensing. Advanced Materials *36*, 2308520. https://doi.org/10.1002/adma.202308520.
5. Volfkovich, Y.M., Bograchev, D.A., Mikhalin, A.A., Rychagov, A.Y., Sosenkin, V.E., and Park, D. (2017). Capacitive deionization of aqueous solutions: modeling and experiments. Desalination and Water Treatment *69*, 130-141. https://doi.org/10.5004/dwt.2017.0469.
6. Wang, H., Varghese, J., and Pilon, L. (2011). Simulation of electric double layer capacitors with mesoporous electrodes: Effects of morphology and electrolyte permittivity. Electrochimica Acta *56*, 6189-6197. https://doi.org/10.1016/j.electacta.2011.03.140.
7. dos Santos, A.P., and Levin, Y. (2015). Electrolytes between dielectric charged surfaces: Simulations and theory. The Journal of Chemical Physics *142*, 194104.

10.1063/1.4921221.

8. Barthel, J., and Buchner, R. (1992). Dielectric permittivity and relaxation of electrolyte solutions and their solvents. Chemical Society Reviews *21*, 263-270.

9. Frisch, M.J., Trucks, G.W., Schlegel, H.B., Scuseria, G.E., Robb, M.A., Cheeseman, J.R., Scalmani, G., Barone, V., Petersson, G.A., Nakatsuji, H., et al. (2019). Gaussian 16 Rev. C.02.

10. Becke, A.D. (1993). Density‐functional thermochemistry. III. The role of exact exchange. The Journal of Chemical Physics *98*, 5648-5652. 10.1063/1.464913.

11. Grimme, S., Ehrlich, S., and Goerigk, L. (2011). Effect of the damping function in dispersion corrected density functional theory. *32*, 1456-1465. https://doi.org/10.1002/jcc.21759.

12. Lu, T., and Chen, F. (2012). Multiwfn: A multifunctional wavefunction analyzer. *33*, 580-592. https://doi.org/10.1002/jcc.22885.

13. Zhang, J., and Lu, T. (2021). Efficient evaluation of electrostatic potential with computerized optimized code. Physical Chemistry Chemical Physics *23*, 20323-20328. 10.1039/D1CP02805G.

14. Berendsen, H.J.C., van der Spoel, D., and van Drunen, R. (1995). GROMACS: A message-passing parallel molecular dynamics implementation. Computer Physics Communications *91*, 43-56. https://doi.org/10.1016/0010-4655(95)00042-E.

15. Martínez, L., Andrade, R., Birgin, E.G., and Martínez, J.M. (2009). PACKMOL: a package for building initial configurations for molecular dynamics simulations. Journal of computational chemistry *30*, 2157-2164. 10.1002/jcc.21224.

16. Wang, J., Wolf, R.M., Caldwell, J.W., Kollman, P.A., and Case, D.A. (2004). Development and testing of a general amber force field. Journal of computational chemistry *25*, 1157-1174. 10.1002/jcc.20035.

17. Lu, T. Sobtop 1.0(dev4) http://sobereva.com/soft/Sobtop (accessed on 8-Jun-2024).

18. Humphrey, W., Dalke, A., and Schulten, K. (1996). VMD: Visual molecular dynamics. Journal of Molecular Graphics *14*, 33-38. https://doi.org/10.1016/0263-7855(96)00018-5.

19. Borodin, O., Olguin, M., Ganesh, P., Kent, P.R.C., Allen, J.L., and Henderson, W.A. (2016). Competitive lithium solvation of linear and cyclic carbonates from quantum chemistry. Physical Chemistry Chemical Physics *18*, 164-175. 10.1039/C5CP05121E.

20. Seo, D.M., Reininger, S., Kutcher, M., Redmond, K., Euler, W.B., and Lucht, B.L. (2015). Role of Mixed Solvation and Ion Pairing in the Solution Structure of Lithium Ion Battery Electrolytes. The Journal of Physical Chemistry C *119*, 14038-14046. 10.1021/acs.jpcc.5b03694.

21. Wu, Y., Wang, A., Hu, Q., Liang, H., Xu, H., Wang, L., and He, X. (2022). Significance of Antisolvents on Solvation Structures Enhancing Interfacial Chemistry in Localized High-Concentration Electrolytes. ACS Central Science *8*, 1290-1298. 10.1021/acscentsci.2c00791.

22. Borba, A., Gómez-Zavaglia, A., Simões, P.N.N.L., and Fausto, R. (2005). Matrix Isolation FTIR Spectroscopic and Theoretical Study of Dimethyl Sulfite. The Journal of Physical Chemistry A *109*, 3578-3586. 10.1021/jp050020t.

23. Salta, Z., Lupi, J., Tasinato, N., Barone, V., and Ventura, O.N. (2020). Unraveling the

role of additional OH-radicals in the H–Abstraction from Dimethyl sulfide using quantum chemical computations. Chemical Physics Letters *739*, 136963. https://doi.org/10.1016/j.cplett.2019.136963.

24. Liu, J., Yuan, B., He, N., Dong, L., Chen, D., Zhong, S., Ji, Y., Han, J., Yang, C., Liu, Y., and He, W. (2023). Reconstruction of LiF-rich interphases through an anti-freezing electrolyte for ultralow-temperature $LiCoO_2$ batteries. Energy & Environmental Science *16*, 1024-1034. 10.1039/D2EE02411J.

25. Pérez-Villar, S., Lanz, P., Schneider, H., and Novák, P. (2013). Characterization of a model solid electrolyte interphase/carbon interface by combined in situ Raman/Fourier transform infrared microscopy. Electrochimica Acta *106*, 506-515. https://doi.org/10.1016/j.electacta.2013.05.124.

26. Li, S., Xu, X., Shi, X., Li, B., Zhao, Y., Zhang, H., Li, Y., Zhao, W., Cui, X., and Mao, L. (2012). Composition analysis of the solid electrolyte interphase film on carbon electrode of lithium-ion battery based on lithium difluoro(oxalate)borate and sulfolane. Journal of Power Sources *217*, 503-508. https://doi.org/10.1016/j.jpowsour.2012.05.114.

27. Xiao, Z., Liu, J., Fan, G., Yu, M., Liu, J., Gou, X., Yuan, M., and Cheng, F. (2020). Lithium bis(oxalate)borate additive in the electrolyte to improve Li-rich layered oxide cathode materials. Materials Chemistry Frontiers *4*, 1689-1696. 10.1039/D0QM00094A.

28. Xu, M., Zhou, L., Hao, L., Xing, L., Li, W., and Lucht, B.L. (2011). Investigation and application of lithium difluoro(oxalate)borate (LiDFOB) as additive to improve the

thermal stability of electrolyte for lithium-ion batteries. Journal of Power Sources *196*, 6794-6801. https://doi.org/10.1016/j.jpowsour.2010.10.050.

29. Zygadło-Monikowska, E., Florjańczyk, Z., Kubisa, P., Biedroń, T., Tomaszewska, A., Ostrowska, J., and Langwald, N. (2010). Mixture of $LiBF_4$ and lithium difluoro(oxalato)borate for application as a new electrolyte for lithium-ion batteries. Journal of Power Sources *195*, 6202-6206. https://doi.org/10.1016/j.jpowsour.2009.10.083.

30. Lu, W., Xie, K., Chen, Z.x., Pan, Y., and Zheng, C.m. (2014). Preparation and characterization of trifluoroethyl aliphatic carboxylates as co-solvents for the carbonate-based electrolyte of lithium-ion batteries. Journal of Fluorine Chemistry *161*, 110-119. https://doi.org/10.1016/j.jfluchem.2014.02.006.

31. Lu, W., Xie, K., Pan, Y., Chen, Z.-x., and Zheng, C.-m. (2013). Effects of carbon-chain length of trifluoroacetate co-solvents for lithium-ion battery electrolytes using at low temperature. Journal of Fluorine Chemistry *156*, 136-143. https://doi.org/10.1016/j.jfluchem.2013.08.015.

32. Choi, S., Choi, K.-W., Kim, S.K., Chung, S., and Lee, S. (2006). Vibrational Structures of Dimethyl Sulfide and Ethylene Sulfide Cations Studied by Vacuum-Ultraviolet Mass-Analyzed Threshold Ionization (MATI) Spectroscopy. The Journal of Physical Chemistry A *110*, 13183-13187. 10.1021/jp0651079.

33. Garton, S.D., Hilton, J., Oku, H., Crouse, B.R., Rajagopalan, K.V., and Johnson, M.K. (1997). Active Site Structures and Catalytic Mechanism of Rhodobacter sphaeroides Dimethyl Sulfoxide Reductase as Revealed by Resonance Raman Spectroscopy.

Journal of the American Chemical Society *119*, 12906-12916. 10.1021/ja972109l.

34. Morita, M., Asai, Y., Yoshimoto, N., and Ishikawa, M. (1998). A Raman spectroscopic study of organic electrolyte solutions based on binary solvent systems of ethylene carbonate with low viscosity solvents which dissolve different lithium salts. Journal of the Chemical Society, Faraday Transactions *94*, 3451-3456. 10.1039/A806278A.

35. Chen, Z., Liu, S., Huang, J., Huang, W., Chen, L., Cui, Y., Du, Y., and Fu, R. (2021). Molecular Level Design of Nitrogen-Doped Well-Defined Microporous Carbon Spheres for Selective Adsorption and Electrocatalysis. ACS Applied Materials & Interfaces *13*, 12025-12032. 10.1021/acsami.1c00002.

36. Chen, J., Zhang, H., Wang, M., Liu, J., Li, C., and Zhang, P. (2016). Improving the electrochemical performance of high voltage spinel cathode at elevated temperature by a novel electrolyte additive. Journal of Power Sources *303*, 41-48. 10.1016/j.jpowsour.2015.10.088.

37. Wu, Z., Li, R., Zhang, S., lv, L., Deng, T., Zhang, H., Zhang, R., Liu, J., Ding, S., Fan, L., et al. (2023). Deciphering and modulating energetics of solvation structure enables aggressive high-voltage chemistry of Li metal batteries. Chem *9*, 650-664. 10.1016/j.chempr.2022.10.027.

38. Zhu, C., Sun, C., Li, R., Weng, S., Fan, L., Wang, X., Chen, L., Noked, M., and Fan, X. (2022). Anion–Diluent Pairing for Stable High-Energy Li Metal Batteries. ACS Energy Letters *7*, 1338-1347. 10.1021/acsenergylett.2c00232.

39. Deng, B., Wang, H., Ge, W., Li, X., Yan, X., Chen, T., Qu, M., and Peng, G. (2017). Investigating the influence of high temperatures on the cycling stability of a

$LiNi_{0.6}Co_{0.2}Mn_{0.2}O_2$ cathode using an innovative electrolyte additive. Electrochimica Acta *236*, 61-71. 10.1016/j.electacta.2017.03.155.

40. Li, Q., Wang, Y., Wang, X., Sun, X., Zhang, J.N., Yu, X., and Li, H. (2020). Investigations on the Fundamental Process of Cathode Electrolyte Interphase Formation and Evolution of High-Voltage Cathodes. ACS Appl Mater Interfaces *12*, 2319-2326. 10.1021/acsami.9b16727.

41. Nohira H, Tsai W, and Besling., W. (2002). Characterization of ALCVD-Al_2O_3 and ZrO_2 layer using X-ray photoelectron spectroscopy. Journal of Non-Crystalline Solids *303*, 83-87.

42. Wang, Q., Yao, Z., Zhao, C., Verhallen, T., Tabor, D.P., Liu, M., Ooms, F., Kang, F., Aspuru-Guzik, A., Hu, Y.-S., et al. (2020). Interface chemistry of an amide electrolyte for highly reversible lithium metal batteries. Nature Communications *11*, 4188. 10.1038/s41467-020-17976-x.

43. Rikka, V.R., Sahu, S.R., Chatterjee, A., Satyam, P.V., Prakash, R., Rao, M.S.R., Gopalan, R., and Sundararajan, G. (2018). In Situ/ex Situ Investigations on the Formation of the Mosaic Solid Electrolyte Interface Layer on Graphite Anode for Lithium-Ion Batteries. The Journal of Physical Chemistry C *122*, 28717-28726. 10.1021/acs.jpcc.8b09210.

44. Zhao, W., Zheng, B., Liu, H., Ren, F., Zhu, J., Zheng, G., Chen, S., Liu, R., Yang, X., and Yang, Y. (2019). Toward a durable solid electrolyte film on the electrodes for Li-ion batteries with high performance. Nano Energy *63*. 10.1016/j.nanoen.2019.06.011.

45. Lu, W., Xiong, S., Pu, W., Xie, K., and Zheng, C. (2017). Carbonate-Grafted Polysilane as a New Additive for Elevated-Temperature Lithium-Ion Batteries. ChemElectroChem *4*, 2012-2018. https://doi.org/10.1002/celc.201700264.

46. Fondard, J., Irisarri, E., Courrèges, C., Palacin, M.R., Ponrouch, A., and Dedryvère, R. (2020). SEI Composition on Hard Carbon in Na-Ion Batteries After Long Cycling: Influence of Salts ($NaPF_6$, NaTFSI) and Additives (FEC, DMCF). Journal of The Electrochemical Society *167*. 10.1149/1945-7111/ab75fd.

47. Philippe, B., Dedryvère, R., Gorgoi, M., Rensmo, H., Gonbeau, D., and Edström, K. (2013). Role of the $LiPF_6$ Salt for the Long-Term Stability of Silicon Electrodes in Li-Ion Batteries—A Photoelectron Spectroscopy Study. Chemistry of Materials *25*, 394-404. 10.1021/cm303399v.

48. Mao, M., Ji, X., Wang, Q., Lin, Z., Li, M., Liu, T., Wang, C., Hu, Y.-S., Li, H., Huang, X., et al. (2023). Anion-enrichment interface enables high-voltage anode-free lithium metal batteries. Nature Communications *14*, 1082. 10.1038/s41467-023-36853-x.
